# Beyond Observation: A Hermeneutic Phenomenological Study of Role Modeling Experiences Among Clinical Residents in an Ethiopian Teaching Hospital: A Qualitative Study

**DOI:** 10.1002/hsr2.73179

**Published:** 2026-09-03

**Authors:** Tamrat Nida, Abdisa Tufa Bedada

**Affiliations:** ^1^ Department of Medical Biochemistry, School of Medicine, College of Health Sciences Addis Ababa University Addis Ababa Ethiopia

**Keywords:** Ethiopia, global health education, hermeneutic phenomenology, hidden curriculum, medical education, professional identity formation, role modeling

## Abstract

**Background and Aims:**

Role modeling is universally regarded as essential to professional identity formation in medical education, yet its experiential and embodied complexity remains underexamined, particularly outside well‐resourced Western settings. This study investigated how clinical residents in an Ethiopian teaching hospital experience role modeling relationships, and how cultural hierarchy, resource scarcity, and postcolonial institutional structures shape that experience.

**Methods:**

We conducted a hermeneutic phenomenological study combining van Manen's life world existential with Interpretative Phenomenological Analysis. Ten purposively sampled residents from five departments at Addis Ababa University completed two rounds of in‐depth interviews three to 4 months apart, 3 months of structured reflective journaling, and were the subject of 20 h of clinical observation by the first author, supplemented by analysis of institutional and personal artifacts, over a 9 month period (September 2024–May 2025).

**Results:**

Role modeling was experienced as an embodied and relational phenomenon organized around four interconnected themes: (1) embodied transmission of clinical knowledge through haptic, kinesthetic, affective, and intercorporeal engagement; (2) temporal disruption of professional becoming through existential hinge points and biographical rupture; (3) dialectical navigation of admiration, resistance, and hierarchical power; and (4) moral formation through witnessing mentors navigate resource constraints, including formation through negative as well as positive modeling.

**Conclusion:**

Role modeling in this Ethiopian residency setting is better understood as a chiasmic, reciprocal intertwining of mentor and resident than as one‐directional transmission. Residents actively interpreted, resisted, and selectively appropriated modeled practices, doing so within a postcolonial institutional context of scarcity and hierarchy that gave this process locally specific form. These findings suggest faculty development, curricula, and institutional policy in comparable resource‐limited settings should explicitly address the embodied and power‐mediated dimensions of modeling.

## Key Points

- Role modeling in clinical education operates through embodied, haptic, and intercorporeal dimensions, not merely visual observation, challenging transmission models that dominate current literature.
- Ethiopian residents actively negotiate, resist, and selectively appropriate modeled practices through sophisticated navigation of cultural hierarchies, resource constraints, and postcolonial institutional structures.
- Pedagogical interventions in resource‐limited settings must address the hidden curriculum's embodied operation and create structured opportunities for ethical witnessing and critical reflection on power dynamics.

## 1 Introduction

### 1.1 The Problem: Role Modeling as a Black Box in Medical Education

Role modeling occupies a paradoxical position in medical education scholarship: universally acknowledged as essential, yet persistently under theorized in its experiential complexity. For decades, researchers have documented the attributes of exemplary role models [1, 2], surveyed students about modeling preferences [3], and advocated for faculty development programs. Yet the dominant research paradigm relying on checklists, surveys, and attribute inventories has systematically obscured the lived experience of being shaped by another clinician's presence, actions, and ways of being.

This gap becomes particularly consequential when we consider two critical observations. First, the mechanisms through which modeling actually works, how embodied presence transforms into professional identity remain largely speculative. Second, the vast majority of role modeling research originates from well resourced, North American, European, and Australian contexts [2, 3], raising fundamental questions about transferability to settings where resource constraints, cultural hierarchies, and postcolonial institutional structures fundamentally reshape educational relationships, a concern anticipated by earlier work on role model selection in culturally diverse, non‐Western medical schools [4] but rarely developed further in the subsequent literature.

This picture is corroborated by recent literature. Koh et al.‘s [5] systematic scoping review of 145 studies on role modeling in professional identity formation found that the field remains dominated by attribute and impact frameworks and called for closer attention to learners’ own sense‐making of modeling experiences. More recent qualitative work has begun to answer that call using designs closer to the lived experience tradition we adopt here: Bransen et al. [6] used a grounded theory approach with Dutch surgical residents to show that modeling operates through discrete, emotionally charged “role model moments” and their negative counterpart, “troll model moments,” rather than through diffuse exposure to admired individuals; and at least two recent studies have applied Interpretative Phenomenological Analysis specifically to role modeling and its adjacent processes in medical education, examining how UK medical students’ identities were shaped by role models along lines of ethnicity [7] and sexual minority status [8]. These studies extend the field beyond attribute checklists toward residents’ and students’ own meaning making, yet all three remain situated in well‐resourced, North American or European settings, and none addresses how resource scarcity or postcolonial institutional hierarchy might reshape the phenomenon they describe. It is precisely this gap that the present study addresses.

### 1.2 Context: Medical Education in Ethiopia

Ethiopia presents a particularly rich context for examining role modeling phenomenologically. Ethiopia has one of the lowest physician to population ratios in sub‐Saharan Africa, a constraint widely documented in health workforce literature on the region ([9]; Federal Ministry of Health of Ethiopia Human Resources for Health reports), and medical education accordingly operates under conditions of profound resource constraint, including high resident to supervisor ratios, intermittent shortages of consumables and equipment, and reliance on a small number of teaching hospitals to train residents across the country [10]. Residency training occurs within hierarchical structures influenced by traditional cultural norms of respect for elders, while simultaneously operating within institutional and curricular structures historically imported from Western medical education models [11]. These conditions scarcity, hierarchy, and cultural hybridity are not merely background variables but actively constitute the phenomenological field within which modeling occurs, a claim we return to analytically in Section Conclusion, 4.

### 1.3 Beyond Transmission: Toward a Phenomenology of Clinical Becoming

The conceptual limitations of existing research stem from its theoretical inheritance. Drawing largely on Bandura's [12] social cognitive theory, role modeling has been conceptualized as unidirectional observational learning, a process wherein novices acquire behaviors by watching experts. This transmission model, while useful for understanding skill acquisition, proves inadequate for grasping how modeling shapes the being of physicians: their ethical sensibilities, their embodied ways of relating to patients, their professional temporality, and their navigation of power.

We propose an alternative theoretical orientation grounded in phenomenological philosophy. Drawing on Merleau‐Ponty's [13, 14] concepts of embodiment and intercorporeality, we conceptualize role modeling not as information transfer but as shared bodily intentionality, a process wherein clinical ways of knowing, valuing, and acting are transmitted through co‐presence in shared spaces of practice. This orientation foregrounds dimensions systematically neglected in the literature: the tactile pedagogy of hands guided through procedures, the affective atmospheres that permeate clinical encounters, the rhythmic organization of work, and the ethical improvisation demanded by resource scarcity.

We draw on Foucault [15] specifically because his account of power departs from a simple top‐down model in which a senior clinician merely possesses and exercises authority over a resident. Foucauldian analysis instead treats power as diffuse, relational, and productive: it operates through everyday practices of observation, correction, and comparison, the ward round that scrutinizes a resident's technique, the informal ranking of who is “good with patients,” the unspoken rules governing when a resident may question a senior colleague, and it produces particular kinds of clinicians rather than only restricting them. Three Foucauldian concepts inform our analysis in Sections 3.3 and 4.3: disciplinary power, the routine surveillance and correction through which clinical competence and conduct are shaped; normalizing judgment, the comparison of residents against an implicit standard of the “good” clinician; and the productive dimension of power, the recognition that hierarchical relationships do not only constrain residents but actively constitute their professional selves. This framework applies with particular force in the setting described in Section 1.2. The steep hierarchies, high resident‐to‐supervisor ratios, and institutional structures inherited from colonial and missionary medical education that characterize Ethiopian residency training are precisely the conditions under which disciplinary power operates most visibly: supervision is frequent and close, deviation from expected conduct is quickly noticed, and cultural norms of respect for seniority further shape what residents feel able to say or resist. Foucauldian analysis, therefore, gives us conceptual purchase on a dimension of role modeling its embeddedness in unequal, disciplinary relationships that transmission models of learning, and even our Merleau‐Pontian account of embodiment, do not on their own adequately address. This theoretical integration allows us to explore how residents navigate, resist, and transform the professional identities offered through modeling within this specific postcolonial institutional context.

We selected these two frameworks deliberately rather than by default. Both were developed to interrogate how bodies are disciplined, trained, and made meaningful within institutions' concerns that travel reasonably well across cultural settings precisely because they do not presuppose any particular content of practice, hierarchy, or value. At the same time, we treat their fit with the Ethiopian context as a question for the analysis rather than an assumption behind it. Both frameworks originate in, and were developed in response to, specific European institutional histories; we therefore use them as interpretive resources to be tested against participants’ accounts, not as templates the data were expected to confirm. Where the data diverged from what these frameworks would predict, for example, where resource scarcity generated forms of ethical improvisation that neither framework directly anticipates, we treat this divergence as analytically significant rather than as a methodological problem to be smoothed over (see Sections 4.4 and 4.8 for explicit discussion of the limits of this theoretical translation).

We clarify here the relationship between theory and analysis that follows from this selection. The decision to approach role modeling phenomenologically, and specifically through van Manen's life world existentials, was made prior to data collection and shaped the interview protocol and journal prompts (Section 2.4), which explicitly invited accounts of embodied, temporal, relational, and spatial experience; in this sense, theory drove the research design. However, the more specific Merleau‐Pontian vocabulary used in presenting the findings of intercorporeality, the chiasm, and kinesthetic mirroring was not specified in advance. It was adopted during Phase 1–2 analysis (Section 2.5), when these concepts proved to capture patterns recurring across participants’ accounts of touch, posture, and synchrony in a way that the original IPA coding labels did not adequately convey. The Foucauldian and infrapolitics vocabulary (Section 4.3) was applied still later, during Phase 4, specifically to interpret patterns of hierarchy and resistance that had already been identified inductively in Phase 3. We therefore distinguish two senses of “theory driven”: phenomenology as a guiding orientation set from the outset, and specific theoretical vocabularies (Merleau‐Ponty, Foucault) applied interpretively once empirical patterns had been identified, rather than imposed on the data from the start. We discuss the limitations of this sequencing, and of these frameworks’ fit to the Ethiopian context, explicitly in Section 4.8.

### 1.4 Research Questions

This study addresses two interconnected questions:

1. How are role models embedded in the lived experience of clinical residents across embodied, temporal, relational, and spatial dimensions such that residents come to interpret, negotiate, and transform modeled practices in their professional becoming?
2. How do cultural norms, resource constraints, and institutional hierarchies shape the phenomenological field within which this modeling occurs?

### 1.5 Significance

This study makes three contributions to medical education scholarship. Methodologically, it demonstrates the application of rigorous phenomenological methods rarely employed in global health education research. Theoretically, it develops a contextualized understanding of role modeling as embodied, temporally complex, and power mediated moving beyond transmission models toward a chiasmic conceptualization of mutual transformation. Practically, it offers evidence‐based insights for faculty development, curriculum design, and institutional policy in resource‐limited settings.

## 2 Methodology

### 2.1 Research Design and Philosophical Orientation

This study employed hermeneutic phenomenology [16, 17], a methodology oriented toward interpreting the meaning structures of lived experience. We positioned ourselves within the constructivist interpretivist paradigm, recognizing that understanding emerges through dialogical engagement between researcher and participant life worlds. The study is reported in accordance with the COREQ checklist for qualitative research (see Supplementary File 1).

### 2.2 Researcher Positionality and Reflexivity

The research team comprised two medical educators: TN (Master's candidate, Medical Education, health science professional background), who conducted all interviews, journal review, and clinical observations, and ATB (PhD, Medical, Assistant professor), who supervised the study and contributed to analytic decisions. TN's interest in this research question arose from his own experience as a recent medical graduate observing wide variation in the quality and ethical character of clinical mentorship during clerkship and internship training, and from a prior academic interest in phenomenological methods developed during his Master's coursework. At the time of data collection, he held prior teaching and supervisory responsibility within the institution. ATB's interest stems from his longer standing program of research on medical research in resource constrained settings and his own educational background in the medical faculty, including over 10 years of experience within Ethiopian medical faculty education. Both authors approached the analysis assuming that role modeling in this setting would likely be shaped by hierarchy and resource scarcity, given their own institutional experience; we treat this as a substantive starting sensitivity that informed our interview guide and journal prompts (Section 1.3) rather than as a bias to be eliminated, consistent with IPA's hermeneutic stance on the researcher's role in meaning making.

TN had no supervisory or evaluative relationship with participants, though he was known as a medical educator within the institution, a position that facilitated participant access and trust while also requiring careful attention to power dynamics, since participants may have moderated accounts of negative modeling experiences out of awareness that the interviewer was institutionally embedded. We addressed this through explicit assurances of confidentiality, by emphasizing that critical accounts were of particular value to the study, and by triangulating interview accounts against journal entries and observational field notes.

Reflexivity was maintained through multiple practices: bracketing interviews before data collection, reflexive journaling throughout, regular peer debriefing, and maintenance of an audit trail of analytical decisions (see Supplementary File 4). Member checking occurred at two levels: transcripts were returned to participants for verification, and preliminary findings were discussed with three participants, whose feedback confirmed interpretive adequacy and refined the articulation of the “Relational Dialectics” theme.

### 2.3 Participants and Setting

Ten clinical residents were purposively selected from five departments at Addis Ababa University School of Medicine, Ethiopia's largest residency training institution. Maximum variation sampling ensured representation across gender, training year, specialty, and prior clinical experience. Table 1 presents participant characteristics.

**Table 1:** Participant demographic and training characteristics.

Participant	Gender	Year	Department	Prior experience	Clinical settings observed
P1	F	R3	Pediatrics	4 years GP	Ward rounds, outpatient
P2	M	R2	Pediatrics	3 years rural	Emergency, neonatal ICU
P3	F	R1	Gynecology	Fresh graduate	OR, labor ward
P4	M	R3	Surgery	5 years diverse	OR, trauma bay
P5	M	R2	Orthopedics	2 years military	Clinic, OR
P6	F	R1	Anesthesia	Fresh graduate	OR, ICU
P7	F	R3	Internal Med	4 years urban	Ward, morning report
P8	M	R2	Family Med	3 years NGO	Community clinic
P9	F	R1	Surgery	Fresh graduate	OR, ward
P10	M	R3	Pediatrics	4 years academic	Subspecialty clinic

Eligibility criteria were: (a) enrollment as a clinical resident (R1–R3) in one of the five participating departments at the time of recruitment; (b) at least 6 months of clinical exposure within the residency program, to ensure participants had had some opportunity for modeling experiences; and (c) willingness to participate in two interview rounds and 3 months of journaling. Residents were approached face to face by the first author following department level introductory presentations of the study, and those expressing interest were given written study information and a minimum of 48 h before providing consent, to reduce any perceived obligation to participate given the first author's institutional visibility.

This sample deliberately spans departments with markedly different clinical cultures, supervisory structures, and procedural intensity (e.g., Anesthesia and Surgery vs. Family Medicine and Pediatrics outpatient settings). Maximum variation sampling was used precisely to surface such differences rather than to obscure them. Where department or seniority appeared to shape the form modeling took, we note this within the relevant theme in Section Results, 3.

### 2.4 Data Collection

Data were collected over 9 months (September 2024–May 2025) through four complementary methods.

#### 2.4.1 Phenomenological Interviews

Two rounds of in depth interviews (60–90 min) were conducted with each participant. First round interviews began with grand tour questions inviting narrative accounts of “moments when you found yourself learning from a senior colleague in ways that felt significant.” Second round interviews were conducted three to 4 months after first round interviews and explored emerging analytic threads identified in the preliminary review of first round data, inviting elaboration on specific modeling episodes. All 10 participants completed both interview rounds; no participant withdrew from the study. Full interview guides, phenomenological probes, and the clinical observation protocol are provided in Supplementary File 2.

#### 2.4.2 Reflective Journals

Participants maintained structured journals for 3 months, responding to weekly phenomenological prompts (e.g., “Describe a moment this week when you became aware of embodying something learned from a role model”). Eight of the ten participants submitted journal entries for at least 10 of the 12 prompted weeks; two participants (P5, P9) submitted entries for 6 and 7 weeks respectively, citing clinical workload. We treated journals as a complementary rather than primary data source given this variable completion (see Section 2.6 and Limitations, Section 4.8). Full journal guidelines and weekly phenomenological prompts provided to participants are presented in Supplementary File 3.

#### 2.4.3 Clinical Observation

The first author conducted 20 h of participant observation across clinical settings, documenting interactions, spatial arrangements, and embodied practices through field notes.

#### 2.4.4 Artifact Analysis

Institutional documents (assessment forms, orientation materials) and participants’ personal artifacts (procedure logs, notes) were analyzed as contextual data.

### 2.5 Data Analysis

Analysis proceeded through four integrated phases, drawing on the full multimodal corpus while assigning each data source a distinct analytic role, summarized in Table 1A below.

**Table 1A:** Contribution of each data source to the analysis.

Data source	Primary analytic role	Phase(s) used
Interview transcripts (Rounds 1–2)	Primary source for idiographic case analysis and identification of Group Experiential Themes (GETs); principal source of illustrative quotations in Section Results, 3	1, 2, 3, 4
Reflective journals	Cross check and elaboration of in the moment embodied/temporal experience; used to corroborate or extend interview accounts, particularly for Theme 1 (embodiment) and Theme 2 (temporality)	2, 3
Clinical observation field notes	Contextualization and corroboration of interview accounts of embodied, spatial, and relational practice; informed researcher sensitivity during interview analysis rather than generating independent themes	1, 3
Institutional and personal artifacts	Contextual/corroborative material situating individual accounts within institutional structures and constraints	3, 4

#### 2.5.1 Phase 1: Phenomenological Reduction

Following van Manen [16], we bracketed theoretical presuppositions through reflexive journaling and maintained an orientation of “wonder” toward the phenomena. Each interview transcript was read multiple times to develop a holistic understanding. Field notes from clinical observation were read alongside interview transcripts at this stage to sensitize the researchers to embodied and spatial dimensions of practice that participants themselves did not always verbalize unprompted.

#### 2.5.2 Phase 2: Lifeworld Existential Analysis

Interview and journal data were organized around van Manen's four lifeworld existentials: lived body (corporality), lived time (temporality), lived space (spatiality), and lived relation (relationality). This provided a systematic framework for identifying experiential structures and was the primary phase in which journal entries, which tended to capture in‐the‐moment embodied and temporal noticing more directly than retrospective interview narration, were drawn upon.

#### 2.5.3 Phase 3: Interpretative Phenomenological Analysis (IPA)

Following Smith et al. [18, 19], Smith and Nizza [20], and current IPA guidance, we conducted line‐by‐line exploratory noting on each interview transcript individually, developing experiential statements and Personal Experiential Themes (PETs) for each participant before identifying Group Experiential Themes (GETs) across cases. This phase began with full idiographic engagement: a complete within case analysis was produced for each of the ten participants individually (presented in full in Supplementary File 4) before any cross case comparison was undertaken, consistent with IPA's idiographic commitment. The four GETs presented in Section Results, 3 represent patterns identified across these individual case analyses, but Section Results, 3 reports illustrative idiographic detail throughout (noting, e.g., where a theme was salient for some participants and not others) rather than treating cross case convergence as the primary analytic goal. Institutional and personal artifacts were used during this phase as contextual material to corroborate or complicate participants’ own accounts (e.g., comparing a participant's account of unsupervised procedural practice against documented supervision policy).

#### 2.5.4 Phase 4: Foucauldian Discourse Analysis

Once the experiential themes above had been established inductively, we returned to the dataset to examine how power relations structured these modeling experiences, identifying disciplinary practices, resistance strategies, and the operation of medical hierarchies (Section 1.3 clarifies the sequencing of this theoretical phase relative to the inductive analysis).

Analysis was iterative, moving between parts and whole, with regular team meetings to challenge interpretations and examine disconfirming evidence.

### 2.6 Idiographic Commitment and the Supplementary Analysis Trail

Readers seeking the full idiographic, case by case analytic trail experiential statements, PETs, exploratory notes, and within case theme development for each of the ten participants are referred to Supplementary File 4, which presents this material in detail. Consistent with current IPA practice, the main text and supplement describe analysis as proceeding from idiographic case analysis to cross‐case patterning, rather than presenting cross‐case thematic description as the starting point.

### 2.7 Transcription and Coding

All interviews were audio recorded with participant consent and transcribed verbatim by the first author (TN), a practice chosen to facilitate deep immersion in the data during the initial analytic phase. All transcripts were subsequently checked against the original audio recordings for accuracy by the supervising author (ATB) to ensure fidelity. Transcription and preliminary coding were conducted in Amharic, the primary language of the interviews and clinical exchanges with salient excerpts and GETs, subsequently translated into English for final analysis and write‐up. To safeguard interpretive accuracy, a back translation process was applied to key passages and core thematic labels, allowing the research team to reconcile any nuances lost in the initial translation. Coding was facilitated using qualitative analysis software (NVivo) for systematic data management, cross case comparison and efficient retrieval of coded segments; this was complemented by manual iterative readings of hard copy transcripts to preserve the holistic, case by case idiographic commitment central to the IPA approach. The full coding framework, analytic decision trail, and iterative revisions of the theme structure were retained and are presented in Supplementary File 4 to ensure transparency and methodological rigor.

### 2.8 Ethical Considerations

The study received approval from the Department of Health Science Education Research and Ethics Review Committee (Ref No. SOM/MEDE/088/2024). Written informed consent was obtained from all participants, with particular attention to confidentiality given the sensitive nature of discussing hierarchical relationships. Participants were assured they could withdraw at any time without consequences.

### 2.9 Statistical and Analytic Software

No inferential statistical testing was performed, as this is a qualitative phenomenological study; participant characteristics in Table 1 are reported descriptively, consistent with guidance that quantitative reporting standards apply only where inferential or descriptive statistical analysis is undertaken. For the management and systematic organization of the coding process, the qualitative data analysis software NVivo (version 14) was employed to facilitate cross‐case comparison, efficient retrieval of coded segments, and the maintenance of the analytic decision trail. As noted in Section 2.7, this software application was used in conjunction with manual iterative readings of hard copy transcripts to preserve the idiographic, case‐sensitive commitment central to the IPA approach.

## 3 Findings

### 3.1 Defining Role Modeling for This Analysis

Before presenting findings, we clarify the analytic boundary between role modeling and other forms of teaching, an ambiguity that warrants explicit clarification given the breadth of the underlying data corpus. We treat an interaction as an instance of role modeling rather than general instruction when the participant's own account indicates that what was learned exceeded the explicit content of the instruction itself: that is, when participants described internalizing something about how the senior clinician was (a disposition, a way of being present, an ethical stance, a rhythm) rather than only what they did or said. Purely procedural guidance (e.g., a supervisor correcting hand position during a task with no further elaboration from the participant) was not coded as role modeling unless the participant explicitly framed the person as a model and described some broader uptake beyond the immediate technical correction. This boundary is necessarily interpretive, consistent with IPA's hermeneutic stance, and we flag in Section 3.2.1 the specific instances where this distinction required careful judgment.

**Figure 1:**
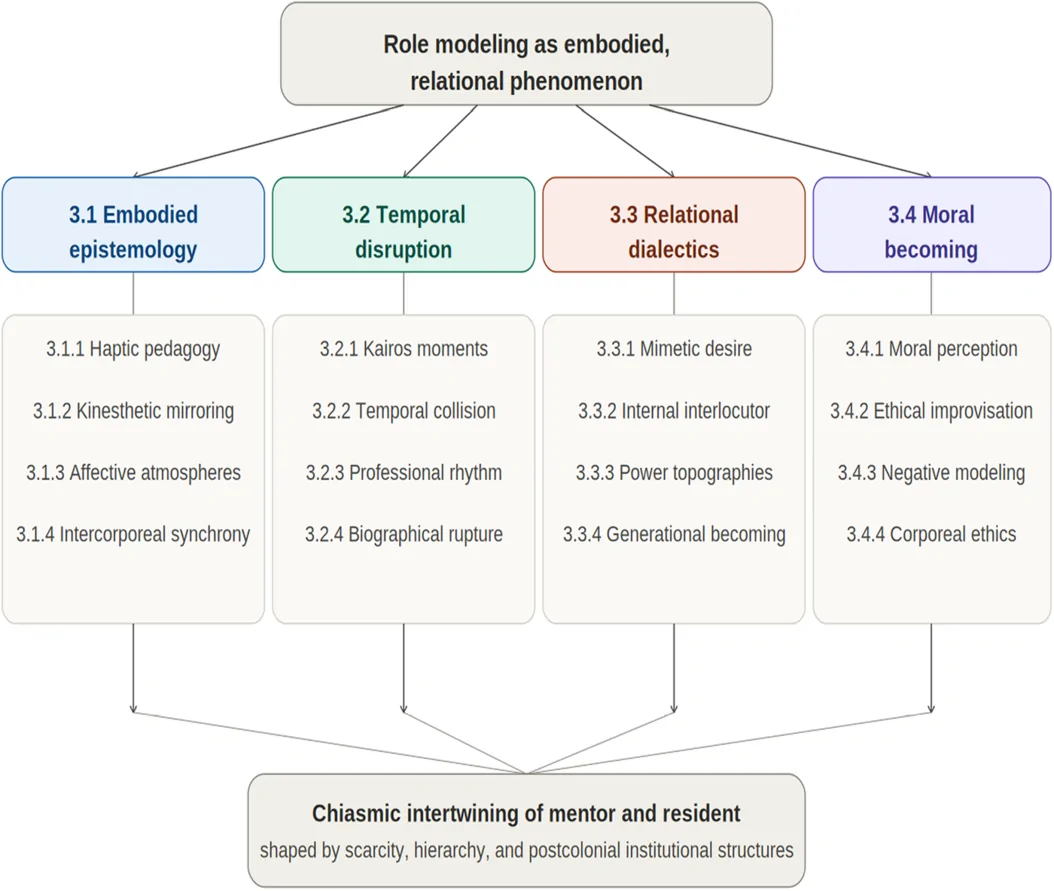
Overview of the four Group Experiential Themes (GETs) and constituent subthemes of role modeling experience.

Figure 1 presents a diagrammatic overview of the four GETs and their constituent subthemes, intended to orient the reader before the detailed narrative below.

Analysis revealed four interconnected GETs that constitute the essential structures of role modeling experience. Each theme is presented with phenomenological description, illustrative participant accounts, and interpretive analysis. Consistent with Section 2.5, we note throughout where a subtheme was salient across most participants versus where it was prominent for a smaller number of participants, in keeping with IPA's idiographic commitment. Notably, we did not identify a systematic pattern whereby earlier year residents (R1) described qualitatively different GETs from later year residents (R3); rather, all four themes were present across training years, though their specific content varied with departmental context, consistent with IPA's idiographic orientation (Section 2.5).

### 3.2 Embodied Epistemology: The Corporeal Transmission of Clinical Knowing

The first theme concerns how clinical knowledge is transmitted not through abstract symbols but through shared bodily presence in clinical spaces. This theme was salient across all ten participants, though its specific subthemes (below) varied by departmental context, with haptic pedagogy most prominent among residents in procedural specialties (Surgery, Gynecology, Orthopedics, and Anesthesia) and affective atmosphere most prominent among residents in ward‐based specialties (Pediatrics, Internal Medicine, and Family Medicine). Residents consistently described learning through modalities that exceed visual observation, engaging the full sensory repertoire of embodied existence.

#### 3.2.1 Haptic Pedagogy: Knowledge Through Touch

Participants emphasized the unique pedagogical power of tactile engagement. This was particularly salient in procedural disciplines but extended beyond technical skill to encompass what might be called “relational touch,” the ways hands convey attention, care, and clinical judgment. We note that the example below was coded as role modeling rather than purely technical instruction because the participant explicitly described an enduring disposition (“my hands remember”) rather than a one‐off correction (see Section Results, 3.0).

When he places his hands over mine during a procedure, I feel the pressure, the angle, the rhythm. That touch teaches more than any lecture. It's like my hands remember what his hands know. Even now, when I'm doing a procedure alone, I sometimes feel his hands guiding mine.

(P4, Surgery)

This haptic dimension created what one participant termed “muscle memory of care,” a corporeal sedimentation of clinical wisdom that persists beyond conscious recall. The pedagogical significance lies in its implicitness: tactile learning occurs below the threshold of verbal articulation, yet fundamentally shapes procedural competence and embodied confidence. Similar accounts of guided touch as a vehicle for tacit transmission of judgment, rather than mere motor correction, were present in interviews with P6, P5, and P9, all from procedural specialties.

#### 3.2.2 Kinesthetic Mirroring: The Body That Remembers

Residents described unconscious processes of bodily imitation that preceded cognitive recognition. Through repeated co‐presence with role models, they found themselves adopting postures, gestures, and rhythms of movement characteristic of their mentors.

I caught myself standing exactly like her at the bedside the same slight lean forward, the same way of holding my hands. My body learned her stance of attentive listening before my mind recognized it. Now I notice I do this when I'm really trying to hear what a patient isn't saying.

(P1, Pediatrics)

This kinesthetic dimension suggests that professional comportment the characteristic ways clinicians inhabit their bodies in clinical space is transmitted through embodied resonance rather than explicit instruction. Clinical observation field notes corroborated this account directly: the first author independently noted P1 adopting a near‐identical bedside posture to her supervising attending during ward rounds observed in month four of data collection, prior to this interview excerpt being collected, supporting the interpretation that this mirroring operates outside of, or prior to, conscious articulation.

#### 3.2.3 Affective Atmospheres: Catching Emotional Dispositions

Beyond specific behaviors, residents described “catching” emotional dispositions from role models. The clinical environment was experienced as suffused with affective qualities that shaped residents’ own emotional experience and regulation.

When he enters the ward, there's a calm that spreads. My own anxiety settles. I've started noticing how my presence affects the team when I'm rushed and anxious, everyone feels it. So I try to carry something of his calm into the room.

(P7, Internal Medicine)

This affective transmission operated through what one participant called “emotional resonance,” a non‐conscious attunement to the emotional states of others that fundamentally shapes clinical presence. We interpret this not simply as emotional contagion in the colloquial sense, but as what affect theorists term a pre‐reflective, relational force that circulates between bodies, spaces, and institutional conditions, and that constitutes rather than merely accompanies professional learning (see Section 4.3 for extended theoretical development of this point). On this reading, P7's account of “carrying” calm forward is not incidental color around a cognitive lesson; the affective atmosphere itself is the pedagogical content, transmitted and reproduced through bodies before it is ever named.

#### 3.2.4 Intercorporeal Synchrony: Shared Bodily Intentionality

The most profound embodied experiences involved moments of what Merleau‐Ponty terms “intercorporeality,” a fusion of bodily intentionality wherein residents experienced themselves as temporarily extended into the body of the other, or as participating in a shared bodily project.

During a resuscitation, our breathing synchronized. I knew what he needed before he asked not because I was thinking, but because my body was somehow moving with his. We weren't two separate people anymore; we were one organism responding to this child's body in crisis.

(P2, Pediatrics)

Table 2 summarizes the embodied dimensions identified in participants’ accounts.

**Table 2:** Embodied dimensions of role modeling experience.

Dimension	Phenomenological description	Representative quote	Pedagogical significance
Proprioceptive	Transmission of postural attitudes and bodily self‐awareness	“My shoulders drop when I emulate his relaxed posture”	Embodied confidence, clinical presence
Haptic	Tactile transmission of skill and care	“Guiding my hand during lumbar puncture”	Procedural calibration, relational touch
Kinesthetic	Adoption of movement patterns and rhythms	“The rhythm of his ward round walk”	Temporal organization of clinical work
Affective	Non‐conscious emotional attunement	“His sigh taught me when to slow down”	Emotional regulation, self‐awareness
Intercorporeal	Shared bodily intentionality in joint action	“Our breathing synchronized during resuscitation”	Team coordination, shared agency

### 3.3 Temporal Disruption: The Phenomenology of Professional Becoming

The second theme concerns how role modeling experiences reorganize residents’ experience of time, their sense of past, present, and future in professional life. This theme illustrates a dynamic that recurs across the dataset: modeling experiences are rarely experienced as discrete, bounded events. Rather, residents repeatedly described moving between memories of earlier training, present clinical encounters, and anticipated future selves as physicians a dynamic resonant with Ibarra's [21] concept of “provisional selves,” and subsequently theorized in medical education as a mechanism by which students and residents use role models to try out possible professional identities [22] such that professional identity appears not as something accumulated additively over time but as something continually revisited, reworked, and carried forward. We develop this interpretive point across the four subthemes below.

#### 3.3.1 Kairos Moments: Existential Hinge Points

Residents identified specific modeling encounters as “kairos moments,” qualitatively distinct experiences wherein ordinary chronological time (chronos) was suspended, and something fundamentally transformative occurred.

I was sitting with her while she told a mother her child had died. Time stopped. I wasn't a resident anymore, wasn't thinking about exams or procedures. I was just present to this moment of human suffering and witnessing how a physician can hold that space. That moment changed me I'm not the same person I was before it.

(P2, Pediatrics)

Before turning to interpretation, it is worth remaining a moment longer with what P2 describes: a resident sitting beside a mother in the instant a child's death is disclosed, exam schedules and procedural anxieties falling away, nothing asked of her except to be present to another person's devastation. There is no technique in this account and no lesson delivered in words; there are only two people in a room, and a resident discovering that she can stay. These moments share phenomenological characteristics: temporal suspension, heightened presence, existential significance, and lasting transformation. They function as “hinge points” around which professional identity reorganizes. We interpret kairos moments such as this one not primarily as instances of learning a clinical skill, but as moments in which a future professional self‐first becomes imaginable to the resident. P2's account is notable for marking a before/after rupture in self‐narrative (“I'm not the same person”) rather than describing the acquisition of new information; what changed was not what P2 knew, but who P2 could now imagine becoming. We treat this distinction between modeling as the transmission of content and modeling as the opening of new identity possibilities as central to this theme.

#### 3.3.2 Temporal Collision: The Simultaneity of Past, Present, and Future

Modeling experiences were frequently described as temporal condensations wherein multiple time horizons converged. Residents experienced themselves simultaneously as novices learning from past models, as present practitioners embodying modeled practices, and as future mentors imagining themselves in their teachers’ positions.

When I'm observing her advocate for a patient, all times exist at once in that observation. I remember my medical school teachers who never advocated, I see what she's doing now, and I imagine myself as an attending doing this for my residents someday. Time folds in on itself.

(P9, Surgery)

This temporal complexity suggests that modeling involves not linear transmission but recursive engagement across multiple temporal registers. P9's account illustrates why we resist describing this as accumulation: the past (absent advocacy in medical school), present (observed advocacy now), and anticipated future (advocacy as attending) are not separate data points being added together, but are experienced together, in a single moment, as mutually defining. The present model is partly constituted by its contrast with the past, and the future self is partly constituted by identification with the present model; each temporal horizon depends on the others for its meaning.

#### 3.3.3 Professional Rhythm: The Temporal Organization of Practice

Through modeling, residents acquired what might be called “professional tempo,” characteristic rhythms of clinical work that organized their experience of time in practice.

Some attending's rush through rounds, and you feel this frantic tempo that makes everyone anxious. Others have a rhythm that somehow includes efficiency AND presence. I've learned a tempo from my mentor a way of moving through the ward that has space for patients but also gets the work done. It's not about speed; it's about rhythm.

(P3, Gynecology)

Negative modeling also shaped temporal experience: residents exposed to chaotic or rushed practice described having to consciously reconstruct alternative temporal organizations.

#### 3.3.4 Biographical Rupture and Continuity

Role modeling experiences could either rupture or consolidate residents’ biographical narratives. Some encounters created sharp discontinuities before/after moments that split professional identity. Others provided threads of continuity, connecting residents to longer traditions of practice.

When I watched him handle that difficult family meeting, something in my story shifted. The person I was before that moment couldn't imagine doing what he did. The person after knows it's possible, even if I'm not there yet.

(P8, Family Medicine)

Taken together, these four subthemes suggest that temporality is not incidental to role modeling but constitutive of it: professional identity formation through modeling is, in an important sense, a temporal accomplishment, achieved through the ongoing re‐weaving of past, present, and anticipated future rather than through the steady accumulation of observed behaviors.

### 3.4 Relational Dialectics: Navigating Admiration and Resistance

The third theme concerns the fundamentally dialectical nature of modeling relationships, the simultaneous pull toward identification and differentiation that characterizes residents’ engagement with role models.

#### 3.4.1 Mimetic Desire and Its Discontents

Participants described a paradoxical structure of wanting to become like their role models while simultaneously rejecting aspects of their being. This created an ongoing dialectic of appropriation and resistance.

I want his surgical skill that exquisite technical precision. But I don't want his arrogance, the way he dismisses nurses, the way patients become procedures. So modeling is this constant negotiation: what to embrace, what to leave behind, what to transform.

(P4, Surgery)

This selective appropriation reveals residents as active interpreters rather than passive recipients of modeled behavior.

#### 3.4.2 The Role Model as Internal Interlocutor

Role models persisted in residents’ consciousness as “internal interlocutors,” imagined presences with whom residents carried on ongoing dialogue, particularly when facing challenging clinical situations.

Even now, when I'm in a difficult situation, I hear her voice. ‘What would Dr. T say here?’ Sometimes I follow what I think she'd do. Sometimes I argue with her. But she's present in my thinking, a constant dialogue partner.

(P7, Internal Medicine)

This internalization suggests that modeling creates enduring relational structures within professional consciousness.

#### 3.4.3 Power Topographies: Navigating Hierarchy

Modeling relationships were embedded within institutional hierarchies that fundamentally shaped their phenomenological structure. Residents navigated complex “power topographies,” uneven terrains of authority, vulnerability, and evaluation.

There are things you can say to some attendings and things you can't. With some, you can ask questions, even disagree respectfully. With others, you learn to perform admiration regardless of what you actually think. Survival requires reading these landscapes accurately.

(P10, Pediatrics)

This strategic navigation reveals the operation of what Scott [23] terms “infrapolitics,” subtle forms of resistance and accommodation within unequal power relations. We note that this is the first point in the findings at which we introduce an explicitly Foucauldian/infrapolitical vocabulary (consistent with the analytic sequencing described in Sections 1.3 and 2.5); we apply it here because participants’ own accounts of “reading the landscape” and “performing admiration” describe precisely the kind of disguised, low visibility resistance Scott's concept names, articulated independently by participants across several departments and training years (P10, P4, P9 in particular) before we identified the conceptual fit.

#### 3.4.4 Generational Becoming: From Resident to Mentor

Residents’ experience of modeling was shaped by their anticipation of becoming models themselves. This temporal orientation created recursive awareness: learning from mentors while simultaneously imagining how they would be for their own future trainees.

I watch him not just to learn, but to learn how to teach. I'm taking notes on what works and what doesn't, building my own model of what kind of attending I want to become. Every interaction is double it's about now and about someday.

(P6, Anesthesia)

### 3.5 Moral Becoming: Ethical Formation Through Embodied Witnessing

The fourth theme concerns how professional ethics are forged not through abstract principles but through embodied engagement with mentors navigating moral complexity.

#### 3.5.1 Moral Perception: Learning to See Ethically

Through modeling, residents developed what might be called “ethical radar,” the capacity to perceive morally salient features of clinical situations. This perceptual learning occurred through observation rather than instruction.

I learned to see what matters ethically by watching her. She'd notice when a patient couldn't afford medication, when a family was being left out of decisions, when a colleague was burned out. Before watching her, I didn't see these things. Now I do.

(P6, Anesthesia)

This finding suggests that moral perception is cultivated through attunement to others’ noticing, not through rule application.

#### 3.5.2 Ethical Improvisation: Phronesis in Practice

Resource constraints in the Ethiopian context demanded what participants described as “ethical improvisation,” the capacity to adapt principles to situations where ideal options are unavailable. This practical wisdom (phronesis) was learned primarily through watching mentors navigate impossible choices.

The textbook says one thing. But we don't have the textbook's resources. I learned ethics by watching him navigate the gap between protocol and what we actually have how to tell a family we can't provide the treatment their child needs, but here's what we CAN do. That's not in any lecture.

(P1, Pediatrics)

This improvisational ethics differs fundamentally from rule‐based approaches, requiring situated judgment developed through modeling. We interpret this finding as evidence of a hidden curriculum specific to conditions of scarcity: residents are not only learning clinical ethics in general, but learning a specific, locally necessary skill, how to preserve a patient's dignity and a family's trust while operating within a resource gap that no amount of textbook ethics addresses. This skill has no equivalent emphasis in role modeling literature developed in well‐resourced settings, and we treat it as one of this study's most context‐specific findings (see Section 4.4).

#### 3.5.3 Negative Modeling and Ethical Witnessing

Paradoxically, negative modeling experiences observing unethical behavior sometimes proved as formative as positive ones, creating what participants called “ethical witnesses”: residents who committed to different practices after witnessing moral failure.

Seeing her humiliate that patient treat her as less than human because she was poor and uneducated made me a witness. I carry that patient's dignity with me. My resistance, my commitment to treat everyone with respect, honors what she suffered in that moment. I became who I am partly because of what I never want to be.

(P3, Gynecology)

P3's account bears sitting with before it is theorized: a colleague reduced, in a single unguarded moment, to her poverty and her lack of education, and a resident forced to watch this happen to someone she could not protect. What P3 carries forward is not a rule but the memory of that patient's face, invoked deliberately, years later, each time P3 is tempted to rush or to dismiss. This finding reveals the complex phenomenology of negative modeling: it can produce not imitation but oppositional identity formation through ethical witnessing.

#### 3.5.4 The Corporeal Ethics of Care

Moral learning extended beyond cognitive judgment to encompass embodied ways of relating to patients. Residents described acquiring what might be called “corporeal ethics,” ethical dispositions carried in posture, touch, and presence.

It's in the way he sits with dying patients not rushing, not avoiding, just present. My body learned that sitting can be a form of care. Now I find myself doing it, not because I decided to, but because my body knows this is what presence looks like.

(P2, Pediatrics)

## 4 Discussion

### 4.1 Beyond Transmission: The Chiasmic Structure of Role Modeling

This study challenges the transmission model that has dominated role modeling research, proposing instead a chiasmic conceptualization. In Merleau‐Ponty's [14] terms, the chiasm names the reversible intertwining of perceiver and perceived, a relation wherein each simultaneously shapes and is shaped by the other. Applied to role modeling, this orientation reveals that:

First, modeling involves reciprocal transformation. While residents are obviously shaped by mentors, our findings reveal multiple directions of influence: residents’ questions and presence shape mentors’ behavior; residents’ selective appropriation transforms modeled practices as they are incorporated into new contexts; and the anticipation of becoming models transforms how residents experience current modeling.

Second, modeling is irreducibly embodied. The dominance of haptic, kinesthetic, affective, and intercorporeal dimensions in participants’ accounts suggests that clinical knowledge is transmitted through shared bodily existence rather than abstract symbol systems. This finding extends phenomenological work in medical education [11, 24] by demonstrating how modeling operates through the prereflective, corporeal dimensions of clinical practice.

Third, modeling involves temporal complexity. The identification of kairos moments, temporal collision, and professional rhythm reveals that modeling reorganizes residents’ experience of time in ways that linear transmission models cannot capture, and as argued in Section 3.2 this temporal reorganization is constitutive of, not incidental to, professional identity formation. This temporal dimension has been largely neglected in role modeling research, yet it appears central to professional identity formation.

### 4.2 The Hidden Curriculum Embodied

Our findings contribute to scholarship on the hidden curriculum [25, 26] by revealing its embodied operation. The hidden curriculum is typically conceptualized as transmitted through institutional practices and informal interactions. Our analysis suggests it is also transmitted through bodies through postures, touches, rhythms, and affective atmospheres that operate below the threshold of explicit awareness.

This embodied hidden curriculum is particularly consequential in resource‐limited settings such as the one studied here, where constant improvisation reveals otherwise invisible values and assumptions. We develop this point further than in the previous version of this manuscript. The ethical improvisation our participants described navigating gaps between protocol and possibility (Section 3.4‐2) is not simply an adaptation residents must make once they arrive in practice; it is itself a curriculum, taught through repeated embodied exposure to mentors making these gaps liveable. Specifically, our data suggest residents learn at least three things through this hidden curriculum of scarcity that are not addressed in role modeling literature developed in well‐resourced settings: first, that the relationship between protocol and possibility is itself a site of ethical judgment, rather than a simple deficiency to be lamented; second, that maintaining a patient's dignity is achievable even when material provision is not, and that mentors model specific embodied and relational strategies (tone, posture, unhurried presence see Section 3.4‐4) for doing so; and third, that improvisation under scarcity is a skill that can itself be performed well or poorly, and that residents learn to evaluate their mentors, and eventually themselves, against this standard. We treat this as a distinctive contribution of this study to hidden curriculum scholarship, rather than as a contextual qualification of findings generated elsewhere.

### 4.3 Affect, Power, and the Infrapolitics of Residency

Before turning to power, we make explicit a theoretical move that was implicit in Section 3.2.3. We understand affect here not merely as individual emotion, but, following recent work in affect theory, as embodied, relational, and often pre‐reflective forces that circulate between people, practices, spaces, and material conditions, shaping professional ways of knowing, feeling, acting, and becoming. On this understanding, the “calm” P7 described catching from a mentor (Section 3.2.3), or the dread residents associated with chaotic ward rhythms (Section 3.2‐3), are not simply emotional reactions that accompany learning; they are part of the medium through which learning and identity formation occur. This affective register operates alongside, and is entangled with, the relations of power discussed below: power in this setting is not only exercised through explicit hierarchy, but through atmospheres of calm, anxiety, and admiration that make certain ways of being a physician feel desirable or unthinkable before any explicit instruction occurs.

Our findings also complicate narratives of residents as passive subjects of professional socialization. Participants demonstrated sophisticated forms of agency within hierarchical constraints: selective appropriation of modeled behaviors, dialogical engagement with internalized mentors, ethical witnessing in response to negative modeling, and strategic navigation of power topographies.

These practices align with Scott's [23] concept of “infrapolitics” the subtle, often invisible forms of resistance that subordinate groups employ within systems of power. Residents’ resistance rarely took the form of open confrontation; instead, it operated through internal differentiation, private commitment to alternative practices, and the construction of counter‐narratives to dominant modeling.

However, this agency remains constrained by institutional structures. The “pedagogical habitus” [27] of this residency program shaped by resource scarcity, cultural hierarchy, and postcolonial institutional forms creates specific conditions for both modeling and resistance. Understanding these conditions is essential for designing interventions that support resident agency without romanticizing it.

### 4.4 Postcolonial Institutional Structures: Toward the Central Contribution of This Study

The postcolonial dimension of this study's contribution is, we now argue, not a peripheral contextual qualifier but its central theoretical payoff. The theoretical frameworks dominating role modeling research, Bandura's social cognitive theory, and even the phenomenological and Foucauldian frameworks we ourselves draw on were developed within, and largely for, well‐resourced Euro‐American institutional settings (Section 1.3). Applying them in an Ethiopian teaching hospital is not a neutral act of theory application; it raises the question of what these frameworks illuminate and what they obscure when the institutional conditions of practice staffing ratios, equipment availability, and curricular inheritance from colonial and missionary medical education (Section 1.2) differ so substantially from the contexts in which the theories were built.

Our findings suggest these frameworks travel imperfectly, and that the imperfection is itself analytically informative. Merleau‐Pontian intercorporeality (Section 3.2.4) and Foucauldian infrapolitics (Section 3.3‐3) describe structures of embodiment and resistance that our data confirm are present in this setting; but neither framework, on its own, anticipates the specific form of ethical improvisation under scarcity that emerged as one of our most distinctive findings (Section 3.4‐2). Mentors in this study were not simply disciplining bodies (Foucault) or sharing bodily intentionality with residents in a context‐neutral sense (Merleau‐Ponty); they were specifically modeling how to practice medicine with integrity inside an institutional structure inherited, in significant part, from elsewhere, and chronically under‐resourced relative to that inherited structure's assumptions. This is the hidden curriculum of postcolonial medical institutions: residents learn not only clinical skill and ethical sensibility in the abstract, but a located form of professional selfhood adequate to practicing within imported institutional forms under conditions those forms did not anticipate.

We therefore propose that future role modeling research in comparable settings treat this postcolonial institutional tension between imported professional and curricular models and local material and cultural conditions as a primary analytic object, rather than as background context. Doing so would extend the chiasmic model proposed in Section 4.1: the “intertwining of perceiver and perceived” in this setting is also, simultaneously, an intertwining of imported and local institutional forms, with residents positioned as active interpreters not only of their individual mentors but of this broader institutional inheritance.

### 4.5 Contextual Specificity and Theoretical Transferability

While this study's findings are deeply contextual shaped by Ethiopian cultural norms, institutional structures, and resource realities they also illuminate dimensions of role modeling likely relevant across contexts. The embodied, temporal, and power‐mediated dimensions we identify may represent structures of modeling experience that recur across settings while taking culturally specific forms locally.

This suggests a methodological principle: rich contextual description enables rather than prevents theoretical transferability to other settings. By understanding how modeling operates in this specific setting, we develop conceptual tools for examining it in others. The chiasmic model we propose, for example, may prove useful for analyzing modeling in other contexts, while requiring contextual specification of how the chiasm operates locally, as Section 4.4 argues it must.

### 4.6 Toward an Integrated Account of Embodiment, Power, and Context

Sections Results, 3 and 4.1 have, of necessity, presented embodiment, temporality, power, and context as analytically separable dimensions of role modeling. We close the discussion by drawing them into closer dialogue, since our data suggest they do not operate as parallel influences on professional becoming but as mutually constituting one another. Hierarchy, for example, is not simply a structure that sits alongside embodied learning and occasionally constrains it; our participants’ accounts suggest hierarchy is itself learned bodily, through the calibrated postures, silences, and forms of address that mark who may touch, correct, or question whom (Section 3.3‐3). Conversely, the embodied dimensions of modeling described in Section 3.1 haptic guidance, kinesthetic mirroring, affective atmosphere are not context neutral channels of transmission that merely happen to occur within a hierarchical, resource constrained institution; they are the specific form hierarchy and scarcity take when they become legible in a resident's body.

A mentor's unhurried, sitting presence with a dying patient (Section 3.4‐4) illustrates the point: it is simultaneously an embodied technique, a moral demonstration, an implicit commentary on what the institution can and cannot provide, and an exercise of the authority to model what “good practice” looks like under these particular material conditions. Reading this moment through embodiment alone, power alone, or scarcity alone would miss what our participants actually seem to describe: a single, dense pedagogical event in which corporeal transmission, ethical formation, hierarchical authority, and material constraint are inseparable aspects of the same lived encounter, and through which postcolonial institutional history becomes, quite literally, incorporated. We suggest that future theorizing of role modeling adopt this interactional stance as a starting assumption treating embodiment, power, context, and moral becoming as facets of a single constitutive process rather than as four themes analyzed in sequence and reassembled afterward, as our own presentation in Section Results, 3 was, for expository reasons, obliged to do.

### 4.7 Implications for Practice

These findings carry implications across four domains faculty development, curriculum design, institutional policy, and resource‐limited settings specifically summarized in Table 3. Current faculty development programs focus heavily on teaching explicit attributes of effective role models; our findings suggest this addresses only the surface of modeling, and that deeper interventions need to engage faculty's embodied, reflective, and power‐related conduct directly. Curricular and institutional interventions, in turn, need to move beyond exposure to good models toward structured reflection, protected space for processing negative modeling, and explicit engagement with the postcolonial tensions discussed in Section 4.4. To make this link explicit, Table 3 organizes the practical implications below directly under the four GETs developed in Section Results, 3 embodied epistemology, temporal disruption, relational dialectics, and moral becoming so that each recommendation is traceable to the specific theoretical work (intercorporeality, temporality, infrapolitics, and phronesis, respectively) that motivates it, rather than appearing as a generic list of domain‐based good practices.

**Table 3:** Implications for practice, organized by theoretical theme.

Theme	Domain	Implication	Suggested action
Embodied Epistemology (Section 3.1)	Faculty development	Cultivate embodied awareness	Introduce a structured 5–10 min post encounter debrief in which faculty and resident jointly review one specific procedural step or patient interaction, using a short observation prompt (e.g., hand positioning, physical proximity during difficult conversations, pacing of speech) so that embodied conduct is named explicitly rather than left for residents to infer
Embodied Epistemology (Section 3.1)	Curriculum design	Create structured observation opportunities	Design clinical experiences maximizing exposure to diverse models, with explicit attention to embodied, haptic, and intercorporeal dimensions of practice
Embodied Epistemology (Section 3.1)	Resource limited settings	Value local knowledge	Recognize contextual, embodied adaptations to scarcity as pedagogical resources warranting systematic study, not merely as workarounds
Temporal Disruption (Section 3.2)	Curriculum design	Integrate reflective practices	Use journals, guided discussion, and narrative exercises timed to kairos moments and hinge points, so that transformative encounters are consolidated rather than lost
Temporal Disruption (Section 3.2)	Faculty development	Develop reflective capacity	Create structured opportunities for faculty to examine their own modeling, including unintended negative influences, across the longer arc of a resident's training
Relational Dialectics (Section 3.3)	Faculty development	Address power dynamics	Support faculty in understanding how hierarchy shapes modeling and in creating space for residents’ productive resistance
Relational Dialectics (Section 3.3)	Institutional policy	Diversify models	Ensure residents encounter varied approaches to practice, reducing dependence on a single model and supporting the selective appropriation described in Section 3.3‐1
Relational Dialectics (Section 3.3)	Institutional policy	Address hierarchical barriers	Examine structures that silence resistance or prevent critical reflection on modeling, including informal channels for raising concerns without reprisal
Moral Becoming (Section 3.4)	Institutional policy	Support ethical witnessing	Create safe spaces for residents to process negative modeling without fear of retribution
Moral Becoming (Section 3.4)	Resource limited settings	Support ethical improvisation	Treat navigation of resource constraints as essential ethical learning a distinct competency to be taught and assessed rather than a regrettable necessity
Moral Becoming (Section 3.4)	Curriculum design	Address the hidden curriculum explicitly	Create forums to critically examine embodied, tacit dimensions of professional formation, including negative as well as positive modeling
Moral Becoming (Section 3.4)	Resource limited settings	Address postcolonial tensions	Explicitly examine tensions between imported professional and curricular models and local practice realities (Section 4.4)

### 4.8 Limitations

This study has several limitations that should inform the interpretation of its findings. As a single institution design, its findings are not intended to be transferable in the statistical sense of generalizability; rather, the rich phenomenological description we provide is intended to enable readers in other settings to judge for themselves how far these findings transfer to their own contexts, a distinction we discuss further in Section 4.5. Because the study examined hierarchical relationships that participants might reasonably hesitate to discuss candidly, social desirability bias may have shaped accounts of negative modeling in particular; we sought to mitigate this through confidentiality assurances, through triangulation across interviews, journals, and observation, and through the first author's explicit signaling that critical accounts were valued, though we cannot rule out residual underreporting. The study also examines only the resident perspective; faculty experiences of being, or attempting to be, role models were not examined, and we see this as an important direction for future research rather than a simple gap. Similarly, all data collection and analysis occurred across the Amharic–English language boundary, and despite careful attention to linguistic and cultural specificity during translation, some experiential nuance may have been lost or altered in this process. Our own positionality as institutional insiders, detailed in Section 2.2, inevitably shaped both data collection and interpretation; we addressed this through systematic reflexivity practices, but readers should weigh our findings with this positionality in view. The 9 month study window allowed us to capture a meaningful span of residency experience, but did not allow us to follow participants into independent practice, where the longer‐term consequences of modeling experiences described here might become apparent. Finally, and reflexively, the theoretical frameworks we apply phenomenological and Foucauldian traditions developed primarily in Western institutional contexts themselves required interpretive translation into this Ethiopian setting; as Section 4.4 argues, we treat the points where this translation proved imperfect as analytically generative rather than as a simple methodological weakness, but readers should recognize that a study designed from the outset around indigenous or context specific theoretical resources might surface dimensions of this phenomenon that our framework does not.

## 5 Conclusion

This phenomenological study reveals role modeling in this Ethiopian medical residency setting as a complex, embodied, temporally organized, and power‐mediated phenomenon fundamentally different from transmission models predominant in the literature. Residents are not passive imitators but active interpreters who selectively appropriate, resist, and transform modeled practices through sophisticated meaning‐making.

The chiasmic structure of modeling the reversible intertwining of perceiver and perceived suggests that professional identity formation occurs not through unidirectional influence but through mutual transformation within relationships structured by institutional hierarchies and cultural contexts. In resource‐limited settings, this process takes specific forms shaped by scarcity, improvisation, and the negotiation of postcolonial institutional forms, a point we have argued (Section 4.4) constitutes this study's central theoretical contribution rather than a contextual footnote to it.

These findings call for pedagogical approaches that recognize the embodied, relational, and power‐mediated nature of modeling. Faculty development must address not only explicit teaching behaviors but also the tacit, corporeal dimensions of clinical presence. Curriculum must create structured opportunities for observing and reflecting on modeling. Institutions must examine how hierarchies enable or constrain productive professional formation.

Ultimately, role modeling represents not merely a teaching strategy but a fundamental dimension of medical becoming, the process through which values are embodied, identities forged, and the future of the profession shaped in the crucible of clinical encounter.

## Author Contributions

**Tamrat Nida:** conceptualization, investigation, writing – original draft, writing – review and editing, methodology, formal analysis, software, data curation. **Abdisa Tufa Bedada:** conceptualization, investigation, funding acquisition, writing – original draft, validation, methodology, visualization, writing – review and editing, formal analysis, project administration, data curation, supervision, resources.

## Funding

The authors have nothing to report.

## Ethics Statement

All authors have read and approved the final version of the manuscript. Abdisa Tufa Bedada, the corresponding author and manuscript guarantor, had full access to all of the data in this study and takes complete responsibility for the integrity of the data and the accuracy of the data analysis.

## Conflicts of Interest

The authors declare no conflicts of interest.

## AI Use in Methodology

Generative AI tools were used in a limited capacity during the preparation of this manuscript. Specifically, an AI assistant was used to assist with the organization and formatting of Supplementary File 4 (the sample analysis trail). The underlying data interview transcripts, reflective journal entries, field observation notes, coding records, and team meeting notes are original research materials generated by the authors. All interpretive analysis, writing, and scholarly decisions were conducted by the authors. No AI tools were used in data collection, transcription, analysis, or generation of participant accounts. This disclosure is made in accordance with the journal's AI disclosure policy and in the interest of full transparency.

## Transparency Statement

Abdisa Tufa Bedada, as manuscript guarantor, affirms that this manuscript is an honest, accurate, and transparent account of the study being reported; that no important aspects of the study have been omitted; and that any discrepancies from the study as planned have been explained.

## Supporting information


Supporting File 1

Supporting File 2

Supporting File 3

Supporting File 4

## Data Availability

The data that support the findings of this study are available on request from the corresponding author. The data are not publicly available due to privacy or ethical restrictions. The data that support the findings of this study are available on request from the corresponding author (abdisa. tufa@aau. edu. et). The data are not publicly available due to privacy and ethical restrictions governing participant confidentiality.
