# Supplementary material for: Beyond Observation: A Hermeneutic Phenomenological Study of Role Modeling Experiences Among Clinical Residents in an Ethiopian Teaching Hospital: A Qualitative Study
Source: Health Sci Rep. 2026 Sep 3;9(9):e73179. doi: 10.1002/hsr2.73179 (PMC13542182; doi:10.1002/hsr2.73179)
Supplement: Supplementary file 1 [file HSR2-9-e73179-s001.docx]

**Supplementary File 3: Reflective Journal Guidelines for Participants**

**A Phenomenological Journal of Role Modeling Experiences**

**Introduction for Participants**

Dear Participant,

Thank you for agreeing to keep this reflective journal as part of our study exploring the lived experience of role modeling during residency. This journal is a space for you to capture moments, reflections, and experiences that might otherwise fade from memory. Your written reflections will provide invaluable insight into how role modeling shapes professional becoming.

This journal is **not** a test or evaluation. There are no right or wrong ways to write. The goal is simply to capture your experience as richly and authentically as possible. Some entries may be lengthy; others may be brief. Some may focus on powerful moments; others on subtle, everyday experiences. All are valuable.

**What Is Phenomenological Reflection?**

Phenomenological reflection means turning our attention to lived experience to what things feel like, look like, sound like, and mean *as we live through them*. Rather than analyzing or judging experiences, we try to describe them in their full sensory and emotional richness.

When writing in this journal, try to:

- **Describe** rather than explain
- **Show** rather than tell
- **Attend to the senses** what you saw, heard, felt in your body, noticed in the space around you
- **Capture moments** rather than generalities
- **Include emotions** without judging them
- **Be specific**—one moment described deeply is worth more than many moments summarized

**Practical Guidelines**

**Duration:** Please write in this journal for **three months** (approximately 12 weeks)

**Frequency:** Aim to write at least **once per week**. Some weeks you may write more often if significant experiences occur.

**Length:** Write as much or as little as feels right. A single paragraph capturing one moment can be as valuable as several pages.

**Timing:** Try to write within 24-48 hours of an experience, while it is still fresh in your memory and felt sense.

**Format:** Use whatever format works for you handwritten in the provided notebook, typed on your computer, or voice-recorded and transcribed. The research team will provide a notebook if you prefer handwriting.

**Confidentiality:** Your journal is confidential. You will have the opportunity to review what you've written and choose what to share. You may also request that certain passages not be used in the research.

**Weekly Phenomenological Prompts**

Each week, you will find a prompt designed to invite reflection on a particular dimension of role modeling experience. You may choose to respond to the weekly prompt **or** write about any other modeling experience that felt significant. The prompts are meant to inspire, not constrain, your reflection.

**Week 1: First Encounters and Ordinary Moments**

**Prompt:** Describe a moment this week when you became aware of learning something large or small from a senior colleague. This could be a dramatic moment or a very ordinary one.

**Guiding Questions:**

- Where were you? Describe the physical space.
- Who was present? Where were they in relation to you?
- What time of day was it? What was the temporal context?
- What specifically did you observe?
- What did you notice in your body at that moment?
- What emotions were present?
- What, if anything, made this moment stay with you?

**Week 2: The Body Remembers**

**Prompt:** Describe a moment when you noticed your body doing something you learned from watching a mentor a posture, a way of moving, a way of touching patients or equipment, a gesture.

**Guiding Questions:**

- What specific bodily action did you notice yourself doing?
- When and where did this happen?
- Can you trace this action back to a specific mentor or modeling moment?
- What did it feel like in your body to perform this action?
- Did you notice it in the moment, or only afterward?
- Was this conscious imitation or something that arose without thinking?
- How does this bodily learning feel different from learning through words?

**Week 3: Emotional Resonance**

**Prompt:** Describe a time when you "caught" or absorbed an emotional state from a mentor calm, anxiety, compassion, frustration, confidence, weariness.

**Guiding Questions:**

- What was the emotional state you experienced?
- Where were you? What was happening?
- How did you become aware of this emotion was it suddenly present, or did it grow gradually?
- Did you notice it in yourself first, or in the mentor?
- What did this emotion feel like in your body?
- How did it affect your actions, thoughts, or presence?
- Did the emotion linger after the moment passed?
- Have you noticed yourself carrying this emotional learning into other situations?

**Week 4: Space and Place**

**Prompt:** Describe a specific clinical space where meaningful modeling occurs for you a particular ward, the OR, a clinic room, a corridor, a morning report room.

**Guiding Questions:**

- Describe this space in detail. What do you see, hear, smell?
- How are bodies arranged in this space during teaching moments?
- Where do mentors typically stand? Where do residents stand?
- How do you feel when you enter this space?
- Are there spaces where modeling feels different more intense, more relaxed, more hierarchical?
- What makes this space conducive (or not conducive) to learning from others?
- How does the space affect what you notice, feel, or learn?

**Week 5: Time and Timing**

**Prompt:** Describe an experience of time during a modeling moment. Did time seem to speed up, slow down, stop, or take on a different quality?

**Guiding Questions:**

- What was happening when your experience of time shifted?
- Can you describe the quality of that altered time?
- Were you aware of the shift as it happened, or only afterward?
- What did this temporal experience allow or enable?
- Did you feel more present, more focused, more aware?
- Have you had other moments with this temporal quality?
- How do these temporal experiences connect with your sense of becoming a physician?

**Week 6: Dialogue and Silence**

**Prompt:** Describe a conversation with a mentor that stood out or a significant moment of silence between you.

**Guiding Questions (for conversation):**

- What prompted the conversation? Where did it happen?
- What was said? What was most memorable?
- What was *not* said that you sensed was present?
- How did you feel during the conversation? Afterward?
- What did this conversation teach you about medicine, about yourself, about your mentor?

**Guiding Questions (for silence):**

- Describe the moment of silence. Where were you? What was happening?
- What was the quality of the silence comfortable, tense, pregnant, empty?
- What did you learn in that silence?
- How did silence communicate what words could not?

**Week 7: Admiration and Ambivalence**

**Prompt:** Describe a moment when you felt both drawn to and critical of something a mentor did or embodied.

**Guiding Questions:**

- What was the situation? What did you observe?
- What aspects drew you in, inspired you, or felt admirable?
- What aspects troubled you, felt wrong, or created discomfort?
- How did you hold these conflicting feelings simultaneously?
- Did you discuss this with anyone? Did you act on either feeling?
- How did this experience affect your relationship with this mentor?
- What did you learn about yourself from this ambivalence?

**Week 8: Ethics in Action**

**Prompt:** Describe a moment when you witnessed a mentor navigating a morally complex or ethically challenging situation.

**Guiding Questions:**

- What was the situation? Who was involved?
- What made it morally complex?
- What did you observe your mentor doing, saying, or embodying?
- What did you notice about how they held themselves posture, voice, gaze, touch?
- What was left uncertain or unresolved?
- What did you learn about ethics from this observation?
- How did this moment affect your sense of what kind of physician you want to become?

**Week 9: Negative Modeling**

**Prompt:** Describe a moment when you observed something in a mentor that you hope never to repeat a behavior, attitude, or way of being that felt wrong or troubling.

**Guiding Questions:**

- What happened? Describe the moment as fully as you can.
- What specifically troubled you?
- How did you feel in the moment? How did you feel afterward?
- Did you respond in any way externally or internally?
- How has this experience stayed with you?
- Has it shaped your commitments or intentions?
- Is there anything you gained from this experience, despite its difficulty?

**Week 10: Becoming a Model**

**Prompt:** Describe a moment when you became aware that a junior colleague—an intern, medical student, or more junior resident—might be learning from observing *you*.

**Guiding Questions:**

- What happened that made you aware of being observed?
- How did this awareness feel?
- What did you notice about your own behavior once you became aware?
- Were there aspects of yourself you hoped they would learn or hoped they would not?
- Did this experience connect you to memories of your own mentors?
- What did this moment teach you about modeling?
- How might this awareness affect your future practice?

**Week 11: Internal Presence**

**Prompt:** Describe a time when you heard a mentor's voice in your head during clinical practice guiding, questioning, supporting, challenging, or comforting.

**Guiding Questions:**

- What was the clinical situation?
- Whose voice arose? What did it say?
- Was this voice helpful, critical, reassuring, or something else?
- Did you find yourself in dialogue with this internal presence?
- How did this internal presence affect your actions or decisions?
- Does this mentor know they live on in you in this way?
- Are there multiple mentors whose voices you carry?

**Week 12: Reflection and Integration**

**Prompt:** Looking back over these three months, what have you learned about how role modeling shapes you? What image, metaphor, or story captures your experience?

**Guiding Questions:**

- Looking back at your journal entries, what patterns do you notice?
- What moments stand out as most significant?
- How would you describe the process of being shaped by role models?
- If you were to create an image or metaphor for your experience, what would it be? (Examples: weaving, being sculpted, absorbing, dialoguing, navigating, growing)
- How has keeping this journal changed your awareness of modeling?
- What do you now understand about role modeling that you didn't before?
- What would you want future residents to know about this dimension of training?

**Additional Prompts for Spontaneous Reflections**

On weeks when you experience a powerful modeling moment that doesn't align with the weekly prompt, feel free to write about that experience using these general questions:

- What happened? Describe the moment in detail.
- Where were you? Who was present?
- What did you notice in your body, emotions, and thoughts?
- What made this moment significant?
- What did you learn about medicine, about yourself, about being a physician?
- How does this moment connect to your past experiences or future aspirations?

**Tips for Deep Phenomenological Writing**

1. **Write in the present tense** occasionally to bring the experience alive: "I am standing at the bedside. His hand touches mine..."
2. **Include sensory details:** What did you see, hear, smell, feel on your skin, notice in your body?
3. **Attend to the in-between:** What happened in the pauses, the transitions, the moments between words and actions?
4. **Notice what surprised you:** Often the most significant learning occurs in moments that defy expectation.
5. **Include your body:** What were your hands doing? Your posture? Your breathing? Your tension or relaxation?
6. **Don't censor:** Write whatever arises, even if it feels incomplete, contradictory, or uncomfortable.
7. **Trust the ordinary:** Profound learning often occurs in moments that seem mundane at the time.

**Confidentiality and Use of Journal Data**

- Your journal is confidential. Only the research team will have access to the full content.
- You will have the opportunity to review your journal entries and indicate any sections you prefer not to be quoted or analyzed.
- When we use quotations in publications, we will remove any identifying information.
- You may withdraw from the study at any time and request that your journal be returned to you or destroyed.

**Final Note**

Thank you for the gift of your reflection. By attending carefully to your own experience and sharing it with us, you are contributing to a deeper understanding of how physicians are formed and ultimately to better education for future generations of Ethiopian doctors.

*"Phenomenology is the study of lived experience the world as we immediately experience it, rather than as we conceptualize or theorize it."*
Max van Manen
