# Supplementary material for: Beyond Observation: A Hermeneutic Phenomenological Study of Role Modeling Experiences Among Clinical Residents in an Ethiopian Teaching Hospital: A Qualitative Study
Source: Health Sci Rep. 2026 Sep 3;9(9):e73179. doi: 10.1002/hsr2.73179 (PMC13542182; doi:10.1002/hsr2.73179)
Supplement: Supplementary file 2 [file HSR2-9-e73179-s002.docx]

**Supplementary File 1: COREQ (Consolidated Criteria for Reporting Qualitative Research) Checklist**

A 32-item checklist for interviews and focus groups

| **Domain/Item** | **Guide Questions/Description** | **Reported in Manuscript** |
| --- | --- | --- |
| **Domain 1: Research Team and Reflexivity** |  |  |
| *Personal Characteristics* |  |  |
| 1. Interviewer/facilitator | Which author/s conducted the interview or focus group? | Section 2.6: "Interviews were conducted by TN (Tamirat Nida)" |
| 2. Credentials | What were the researcher's credentials? (e.g., PhD, MD) | Section 2.6: "[M.Sc](https://m.sc/) in Medical Education" (TN); "PhD" (ATB) |
| 3. Occupation | What was their occupation at the time of the study? | Section 2.6: "Master's candidate in Medical Education" (TN); "medical educator" (ATB) |
| 4. Gender | Was the researcher male or female? | Section 2.6: "two males" |
| 5. Experience and training | What experience or training did the researcher have? | Section 2.6: "advanced training in qualitative methodology and experience conducting three previous phenomenological studies" |
| *Relationship with Participants* |  |  |
| 6. Relationship established | Was a relationship established prior to study commencement? | Section 2.6: "The interviewer had no prior clinical or supervisory relationship with participants" |
| 7. Participant knowledge of interviewer | What did participants know about the researcher? (e.g., personal goals, reasons for doing the research) | Section 2.6: "Participants were informed that the researcher was a medical educator interested in improving residency training through understanding lived experiences" |
| 8. Interviewer characteristics | What characteristics were reported about the interviewer/facilitator? (e.g., bias, assumptions, reasons for interest in the topic) | Section 2.6: "insider within the institution (but outsider to clinical departments)"; reflexive journaling; bracketing of assumptions |
| **Domain 2: Study Design** |  |  |
| *Theoretical Framework* |  |  |
| 9. Methodological orientation and theory | What methodological orientation was stated to underpin the study? (e.g., grounded theory, discourse analysis, ethnography, phenomenology, content analysis) | Section 2.1: "hermeneutic phenomenological approach (van Manen, 1990)"; "constructivist interpretivist paradigm" |
| *Participant Selection* |  |  |
| 10. Sampling | How were participants selected? (e.g., purposive, convenience, consecutive, snowball) | Section 2.2: "purposively selected"; "maximum variation sampling" |
| 11. Method of approach | How were participants approached? (e.g., face-to-face, telephone, mail, email) | Not explicitly stated in manuscript (assumed face-to-face recruitment through departments) |
| 12. Sample size | How many participants were in the study? | Section 2.2: "Ten clinical residents" |
| 13. Non-participation | How many people refused to participate or dropped out? Reasons? | Not reported (assumed all approached agreed to participate) |
| *Setting* |  |  |
| 14. Setting of data collection | Where was the data collected? (e.g., home, clinic, workplace) | Section 2.3: Interviews conducted in clinical settings; observations across "ward rounds, outpatient, emergency, neonatal ICU, OR, labor ward, trauma bay, clinic, ICU, morning report, community clinic, subspecialty clinic" |
| 15. Presence of non-participants | Was anyone else present besides the participants and researchers? | Not explicitly stated |
| 16. Description of sample | What are the important characteristics of the sample? (e.g., demographic data, date) | Section 2.2, Table 1: Gender, year, department, prior experience, modeling relationships described |
| *Data Collection* |  |  |
| 17. Interview guide | Were questions, prompts, guides provided by the authors? Was it pilot tested? | Section 2.3: "grand tour questions regarding moments of conscious learning from senior clinicians"; full guide referenced as Supplementary File 2 |
| 18. Repeat interviews | Were repeat interviews carried out? If yes, how many? | Section 2.3: "Two rounds of in-depth interviews" |
| 19. Audio/visual recording | Did the research use audio or visual recording to collect the data? | Implicitly yes (interviews recorded and transcribed) |
| 20. Field notes | Were field notes made during and/or after the interview or focus group? | Section 2.3: "Researcher field notes were compiled from 20 hours of clinical observation" |
| 21. Duration | What was the duration of the interviews or focus group? | Section 2.3: "60-90 minutes" |
| 22. Data saturation | Was data saturation discussed? | Not explicitly stated; phenomenological research emphasizes depth over saturation |
| 23. Transcripts returned | Were transcripts returned to participants for comment and/or correction? | Section 2.6: "Transcripts were returned to participants for member checking; minor clarifications were incorporated" |
| **Domain 3: Analysis and Findings** |  |  |
| *Data Analysis* |  |  |
| 24. Number of data coders | How many data coders coded the data? | Two |
| 25. Description of the coding tree | Did authors provide a description of the coding tree? | Themes described in Section 3 with subthemes; full coding tree available in Supplementary File 4 |
| 26. Derivation of themes | Were themes identified in advance or derived from the data? | Section 2.4: "derived from data" through phenomenological analysis |
| 27. Software | What software, if applicable, was used to manage the data? | Not specified |
| 28. Participant checking | Did participants provide feedback on the findings? | Section 2.6: "Preliminary findings were shared with three participants for member checking; their feedback confirmed interpretive adequacy and led to minor refinements" |
| *Reporting* |  |  |
| 29. Quotations presented | Were participant quotations presented to illustrate the themes/findings? Was each quotation identified? (e.g., participant number) | Section 3: Extensive quotations with participant identifiers (e.g., P1, P4) |
| 30. Data and findings consistent | Was there consistency between the data presented and the findings? | Yes – themes directly supported by quotations |
| 31. Clarity of major themes | Were major themes clearly presented in the findings? | Section 3: Four Group Experiential Themes (GETs) clearly articulated with subthemes |
| 32. Clarity of minor themes | Is there a description of diverse cases or discussion of minor themes? | Included within each theme (e.g., negative modeling, resistance strategies) |

**Declaration:** This COREQ checklist has been completed to ensure comprehensive and transparent reporting of the qualitative research presented in the manuscript "Beyond Observation: A Hermeneutic Phenomenological Study of Role Modeling Experiences Among Clinical Residents in Ethiopia."

**Reference for COREQ:**
Tong A, Sainsbury P, Craig J. Consolidated criteria for reporting qualitative research (COREQ): a 32-item checklist for interviews and focus groups. *International Journal for Quality in Health Care*. 2007;19(6):349-357.
