# Supplementary material for: Beyond Observation: A Hermeneutic Phenomenological Study of Role Modeling Experiences Among Clinical Residents in an Ethiopian Teaching Hospital: A Qualitative Study
Source: Health Sci Rep. 2026 Sep 3;9(9):e73179. doi: 10.1002/hsr2.73179 (PMC13542182; doi:10.1002/hsr2.73179)
Supplement: Supplementary file 3 [file HSR2-9-e73179-s003.docx]

**Supplementary File 3: Reflective Journal Guidelines for Participants**

**A Phenomenological Journal of Role Modeling Experiences**

**Introduction for Participants**

Dear Participant,

Thank you for agreeing to keep this reflective journal as part of our study exploring the lived experience of role modeling during residency. This journal is a space for you to capture moments, reflections, and experiences that might otherwise fade from memory. Your written reflections will provide invaluable insight into how role modeling shapes professional becoming.

This journal is not a test or evaluation. There are no right or wrong ways to write. The goal is simply to capture your experience as richly and authentically as possible. Some entries may be lengthy; others may be brief. Some may focus on powerful moments; others on subtle, everyday experiences. All are valuable.

**What Is Phenomenological Reflection?**

Phenomenological reflection means turning our attention to lived experience to what things feel like, look like, sound like, and mean *as we live through them*. Rather than analyzing or judging experiences, we try to describe them in their full sensory and emotional richness.

When writing in this journal, try to:

- **Describe** rather than explain
- **Show** rather than tell
- **Attend to the senses** what you saw, heard, felt in your body, noticed in the space around you
- **Capture moments** rather than generalities
- **Include emotions** without judging them
- **Be specific** one moment described deeply is worth more than many moments summarized

**Practical Guidelines**

**Duration:** Please write in this journal for three months (approximately 12 weeks)

**Frequency:** Aim to write at least once per week. Some weeks you may write more often if significant experiences occur.

**Length:** Write as much or as little as feels right. A single paragraph capturing one moment can be as valuable as several pages.

**Timing:** Try to write within 24-48 hours of an experience, while it is still fresh in your memory and felt sense.

**Format:** Use whatever format works for you handwritten in the provided notebook, typed on your computer, or voice-recorded and transcribed. The research team will provide a notebook if you prefer handwriting.

**Confidentiality:** Your journal is confidential. You will have the opportunity to review what you've written and choose what to share. You may also request that certain passages not be used in the research.

**Weekly Phenomenological Prompts**

Each week, you will find a prompt designed to invite reflection on a particular dimension of role modeling experience. You may choose to respond to the weekly prompt **or** write about any other modeling experience that felt significant. The prompts are meant to inspire, not constrain, your reflection.

**Week 1: First Encounters and Ordinary Moments**

**Prompt:** Describe a moment this week when you became aware of learning something large or small from a senior colleague. This could be a dramatic moment or a very ordinary one.

**Guiding Questions:**

- Where were you? Describe the physical space.
- Who was present? Where were they in relation to you?
- What time of day was it? What was the temporal context?
- What specifically did you observe?
- What did you notice in your body at that moment?
- What emotions were present?
- What, if anything, made this moment stay with you?

**Week 2: The Body Remembers**

**Prompt:** Describe a moment when you noticed your body doing something you learned from watching a mentor a posture, a way of moving, a way of touching patients or equipment, a gesture.

**Guiding Questions:**

- What specific bodily action did you notice yourself doing?
- When and where did this happen?
- Can you trace this action back to a specific mentor or modeling moment?
- What did it feel like in your body to perform this action?
- Did you notice it in the moment, or only afterward?
- Was this conscious imitation or something that arose without thinking?
- How does this bodily learning feel different from learning through words?

**Week 3: Emotional Resonance**

**Prompt:** Describe a time when you "caught" or absorbed an emotional state from a mentor calm, anxiety, compassion, frustration, confidence, weariness.

**Guiding Questions:**

- What was the emotional state you experienced?
- Where were you? What was happening?
- How did you become aware of this emotion was it suddenly present, or did it grow gradually?
- Did you notice it in yourself first, or in the mentor?
- What did this emotion feel like in your body?
- How did it affect your actions, thoughts, or presence?
- Did the emotion linger after the moment passed?
- Have you noticed yourself carrying this emotional learning into other situations?

**Week 4: Space and Place**

**Prompt:** Describe a specific clinical space where meaningful modeling occurs for you a particular ward, the OR, a clinic room, a corridor, a morning report room.

**Guiding Questions:**

- Describe this space in detail. What do you see, hear, smell?
- How are bodies arranged in this space during teaching moments?
- Where do mentors typically stand? Where do residents stand?
- How do you feel when you enter this space?
- Are there spaces where modeling feels different more intense, more relaxed, more hierarchical?
- What makes this space conducive (or not conducive) to learning from others?
- How does the space affect what you notice, feel, or learn?

**Week 5: Time and Timing**

**Prompt:** Describe an experience of time during a modeling moment. Did time seem to speed up, slow down, stop, or take on a different quality?

**Guiding Questions:**

- What was happening when your experience of time shifted?
- Can you describe the quality of that altered time?
- Were you aware of the shift as it happened, or only afterward?
- What did this temporal experience allow or enable?
- Did you feel more present, more focused, more aware?
- Have you had other moments with this temporal quality?
- How do these temporal experiences connect with your sense of becoming a physician?

**Week 6: Dialogue and Silence**

**Prompt:** Describe a conversation with a mentor that stood out or a significant moment of silence between you.

**Guiding Questions (for conversation):**

- What prompted the conversation? Where did it happen?
- What was said? What was most memorable?
- What was *not* said that you sensed was present?
- How did you feel during the conversation? Afterward?
- What did this conversation teach you about medicine, about yourself, about your mentor?

**Guiding Questions (for silence):**

- Describe the moment of silence. Where were you? What was happening?
- What was the quality of the silence comfortable, tense, pregnant, empty?
- What did you learn in that silence?
- How did silence communicate what words could not?

**Week 7: Admiration and Ambivalence**

**Prompt:** Describe a moment when you felt both drawn to and critical of something a mentor did or embodied.

**Guiding Questions:**

- What was the situation? What did you observe?
- What aspects drew you in, inspired you, or felt admirable?
- What aspects troubled you, felt wrong, or created discomfort?
- How did you hold these conflicting feelings simultaneously?
- Did you discuss this with anyone? Did you act on either feeling?
- How did this experience affect your relationship with this mentor?
- What did you learn about yourself from this ambivalence?

**Week 8: Ethics in Action**

**Prompt:** Describe a moment when you witnessed a mentor navigating a morally complex or ethically challenging situation.

**Guiding Questions:**

- What was the situation? Who was involved?
- What made it morally complex?
- What did you observe your mentor doing, saying, or embodying?
- What did you notice about how they held themselves posture, voice, gaze, touch?
- What was left uncertain or unresolved?
- What did you learn about ethics from this observation?
- How did this moment affect your sense of what kind of physician you want to become?

**Week 9: Negative Modeling**

**Prompt:** Describe a moment when you observed something in a mentor that you hope never to repeat a behavior, attitude, or way of being that felt wrong or troubling.

**Guiding Questions:**

- What happened? Describe the moment as fully as you can.
- What specifically troubled you?
- How did you feel in the moment? How did you feel afterward?
- Did you respond in any way externally or internally?
- How has this experience stayed with you?
- Has it shaped your commitments or intentions?
- Is there anything you gained from this experience, despite its difficulty?

**Week 10: Becoming a Model**

**Prompt:** Describe a moment when you became aware that a junior colleague an intern, medical student, or more junior resident might be learning from observing *you*.

**Guiding Questions:**

- What happened that made you aware of being observed?
- How did this awareness feel?
- What did you notice about your own behavior once you became aware?
- Were there aspects of yourself you hoped they would learn or hoped they would not?
- Did this experience connect you to memories of your own mentors?
- What did this moment teach you about modeling?
- How might this awareness affect your future practice?

**Week 11: Internal Presence**

**Prompt:** Describe a time when you heard a mentor's voice in your head during clinical practice guiding, questioning, supporting, challenging, or comforting.

**Guiding Questions:**

- What was the clinical situation?
- Whose voice arose? What did it say?
- Was this voice helpful, critical, reassuring, or something else?
- Did you find yourself in dialogue with this internal presence?
- How did this internal presence affect your actions or decisions?
- Does this mentor know they live on in you in this way?
- Are there multiple mentors whose voices you carry?

**Week 12: Reflection and Integration**

**Prompt:** Looking back over these three months, what have you learned about how role modeling shapes you? What image, metaphor, or story captures your experience?

**Guiding Questions:**

- Looking back at your journal entries, what patterns do you notice?
- What moments stand out as most significant?
- How would you describe the process of being shaped by role models?
- If you were to create an image or metaphor for your experience, what would it be? (Examples: weaving, being sculpted, absorbing, dialoguing, navigating, growing)
- How has keeping this journal changed your awareness of modeling?
- What do you now understand about role modeling that you didn't before?
- What would you want future residents to know about this dimension of training?

**Additional Prompts for Spontaneous Reflections**

On weeks when you experience a powerful modeling moment that doesn't align with the weekly prompt, feel free to write about that experience using these general questions:

- What happened? Describe the moment in detail.
- Where were you? Who was present?
- What did you notice in your body, emotions, and thoughts?
- What made this moment significant?
- What did you learn about medicine, about yourself, about being a physician?
- How does this moment connect to your past experiences or future aspirations?

**Tips for Deep Phenomenological Writing**

1. **Write in the present tense** occasionally to bring the experience alive: "I am standing at the bedside. His hand touches mine..."
2. **Include sensory details:** What did you see, hear, smell, feel on your skin, notice in your body?
3. **Attend to the in-between:** What happened in the pauses, the transitions, the moments between words and actions?
4. **Notice what surprised you:** Often the most significant learning occurs in moments that defy expectation.
5. **Include your body:** What were your hands doing? Your posture? Your breathing? Your tension or relaxation?
6. **Don't censor:** Write whatever arises, even if it feels incomplete, contradictory, or uncomfortable.
7. **Trust the ordinary:** Profound learning often occurs in moments that seem mundane at the time

**Confidentiality and Use of Journal Data**

- Your journal is confidential. Only the research team will have access to the full content.
- You will have the opportunity to review your journal entries and indicate any sections you prefer not to be quoted or analyzed.
- When we use quotations in publications, we will remove any identifying information.
- You may withdraw from the study at any time and request that your journal be returned to you or destroyed.

**Final Note**

Thank you for the gift of your reflection. By attending carefully to your own experience and sharing it with us, you are contributing to a deeper understanding of how physicians are formed and ultimately to better education for future generations of Ethiopian doctors.

*"Phenomenology is the study of lived experience the world as we immediately experience it, rather than as we conceptualize or theorize it."*
 Max van Manen

**End of Supplementary File 3**

Supplementary File 4: Sample Analysis Trail

OK, the user has provided the full manuscript and three supplementary files, and now they want the fourth supplementary file: the sample analysis trail. Looking at the history, this is a meticulous researcher who has revised their manuscript based on rejections and is now preparing all the supplementary materials for a high-impact journal submission. They've already done the COREQ checklist, interview protocol, and journal guidelines. The analysis trail is the last piece they need to complete the package.

The user's surface need is clear: they need a professionally formatted analysis trail document. But their deeper needs are probably about demonstrating methodological rigor and transparency to overcome previous rejections. High-impact journals want to see that the qualitative analysis was systematic, trustworthy, and not just a bunch of quotes thrown together. They want to see the "audit trail" the evidence that the themes genuinely emerged from the data through a credible process.

The user is likely the first author, who is a Master's candidate but has experience. They need this document to show reviewers that despite being early career, they conducted analysis at a high standard. The trail needs to show the step-by-step process, the coding evolution, the team discussions, and the reflexive practices. It should bridge the gap between raw data and final themes.

The assistant's response is comprehensive. It structures the trail logically: overview, data excerpts, coding evolution, audit trail, theme development, reflexivity, final thematic structure. This mirrors best practices for qualitative appendices. The thinking process would involve deciding what elements are most convincing to include. The assistant likely thought: "I need to show raw data, show how we moved from codes to themes, show that we debated and refined interpretations, and show that we were reflexive. I should pick excerpts that vividly illustrate the main themes from the paper (embodied epistemology, temporal disruption, etc.) to create a clear link. I should also include a visual (the thematic map) and the audit log to make the process tangible."

The assistant also cleverly includes a "coding evolution table" to show the iterative nature of analysis, and excerpts from reflexive journals to address researcher positionality. This directly speaks to quality criteria in qualitative research. The final part connects each theme back to the data excerpts and provides a cross-reference table to the manuscript, making it easy for reviewers to check the evidence. This shows the assistant understood the user's need for a document that serves as both a record and a persuasive piece of evidence for the journal reviewers.

**Supplementary File 4: Sample Analysis Trail**

**A Phenomenological Journey from Data to Interpretation**

**Introduction to the Analysis Trail**

This document provides a transparent account of the analytical journey from raw interview data to final thematic interpretations. Drawing on principles of hermeneutic phenomenology and Interpretative Phenomenological Analysis (IPA), we present a step-by-step illustration of how one core theme **Embodied Epistemology** emerged and evolved through iterative engagement with participant accounts.

This trail demonstrates:

- Initial encounters with raw data
- Line-by-line coding processes
- Development of Personal Experiential Themes (PETs)
- Integration across participants
- Movement toward Group Experiential Themes (GETs)
- The role of theoretical frameworks in interpretation
- Reflexive considerations throughout analysis

**Part A: Data Excerpt with Initial Coding**

**Raw Data Extract: Interview with P4 (Surgery)**

*Context: P4, a third-year surgery resident, is describing a pivotal learning moment with his attending surgeon during a complex procedure.*

**Interview Transcript (Excerpt)**

**TN:** You mentioned a specific moment when your attending guided your hands during a procedure. Can you take me back to that moment?

**P4:** Yes. It was during a Whipple procedure very complex, very tense. I was struggling with a particular anastomosis, my hands weren't finding the right angle, the right tension. Dr. T was across from me, and without a word, he reached over and placed his hands over mine. Not gripping, not controlling just... present. I felt the pressure change, the slight adjustment in angle, the rhythm of how he moves through tissue. For a few seconds, we were moving together. My hands weren't mine alone anymore; they were somehow his hands too, or maybe our hands together.

| Line | Data Segment | Initial Code |
| --- | --- | --- |
| 1 | "During a Whipple procedure very complex, very tense" | High-stakes context; procedural complexity |
| 2 | "I was struggling with a particular anastomosis" | Moment of difficulty; learner vulnerability |
| 3 | "My hands weren't finding the right angle, the right tension" | Tactile uncertainty; proprioceptive struggle |
| 4 | "Dr. T was across from me" | Spatial positioning; face-to-face arrangement |
| 5 | "Without a word, he reached over" | Non-verbal intervention; silence as pedagogy |
| 6 | "Placed his hands over mine" | Tactile contact; haptic teaching |
| 7 | "Not gripping, not controlling just... present" | Quality of touch; non-dominating presence |
| 8 | "I felt the pressure change, the slight adjustment in angle" | Tactile perception; haptic learning |
| 9 | "The rhythm of how he moves through tissue" | Temporal dimension; rhythm; embodied style |
| 10 | "For a few seconds, we were moving together" | Shared movement; synchrony; intercorporeality |
| 11 | "My hands weren't mine alone anymore" | Boundary dissolution; extended embodiment |
| 12 | "They were somehow his hands too, or maybe our hands together" | Collective agency; shared intentionality |
| 13 | "Everything slowed down" | Temporal alteration; kairos moment |
| 14 | "My breathing, which had been shallow and fast, deepened" | Somatic shift; physiological attunement |
| 15 | "I felt my shoulders drop" | Release of tension; embodied relaxation |
| 16 | "I hadn't realized I'd been holding them up by my ears" | Previously unaware tension; somatic awareness |
| 17 | "There was warmth where his hands touched mine" | Sensory quality; affective dimension of touch |
| 18 | "I could feel what he was feeling through his hands" | Empathetic touch; sensory transfer |
| 19 | "The resistance of the tissue, the right amount of tension" | Tactile discernment; embodied judgment |
| 20 | "My hands became extensions of his judgment" | Tool-like extension; distributed cognition |
| 21 | "When he lifted his hands, something had changed" | Post-intervention transformation |
| 22 | "My hands *knew* something they hadn't known before" | Bodily knowledge; somatic learning |
| 23 | "Not in my head—I couldn't have explained it in words" | Non-cognitive; pre-reflective; tacit knowledge |
| 24 | "But in my fingers, in my wrists, in the way I held myself" | Corporeal localization; embodied memory |
| 25 | "Even now, months later, when I do that anastomosis" | Temporal persistence; enduring embodiment |
| 26 | "I sometimes feel his hands over mine" | Phantom presence; internalized mentor |
| 27 | "The knowledge is in my body" | Somatic epistemology; embodied knowing |
| 28 | "A textbook tells you steps. A lecture gives you principles." | Contrast with formal learning |
| 29 | "This was my body learning from his body, directly" | Intercorporeal transmission |
| 30 | "Without words getting in the way" | Beyond language; pre-verbal learning |
| 31 | "Like the difference between reading about how to ride a bicycle" | Analogy; procedural embodiment |
| 32 | "Having someone steady the seat while you find your balance" | Scaffolded learning; supported practice |
| 33 | "Your body just *gets* it" | Somatic comprehension; embodied understanding |

**TN:** What did you notice in your body during those seconds?

**P4:** Everything slowed down. My breathing, which had been shallow and fast, deepened. I felt my shoulders drop I hadn't realized I'd been holding them up by my ears. There was warmth where his hands touched mine. And then this strange thing: I could feel what he was feeling through his hands the resistance of the tissue, the right amount of tension. It was like my hands became extensions of his judgment.

**TN:** And after those seconds?

**P4:** When he lifted his hands, something had changed. My hands *knew* something they hadn't known before. Not in my head I couldn't have explained it in words but in my fingers, in my wrists, in the way I held myself. Even now, months later, when I do that anastomosis, I sometimes feel his hands over mine. The knowledge is in my body.

**TN:** How is that different from other kinds of learning?

**P4:** Completely different. A textbook tells you steps. A lecture gives you principles. But this... this was my body learning from his body, directly, without words getting in the way. It's like the difference between reading about how to ride a bicycle and having someone steady the seat while you find your balance. Your body just *gets* it.

**Initial Line-by-Line Coding**

**Part B: Developing Personal Experiential Themes**

**Clustering Initial Codes into Personal Experiential Themes (P4 Only)**

After coding the full P4 transcript, initial codes were clustered into Personal Experiential Themes:

**Cluster 1: Tactile Pedagogy**

- Non-verbal intervention
- Tactile contact
- Quality of touch
- Haptic learning
- Tactile perception
- Empathetic touch
- Tactile discernment

**Personal Experiential Theme 1A: Hands That Teach** *The pedagogical power of direct tactile engagement*

**Cluster 2: Bodily Transformation**

- Somatic shift
- Release of tension
- Previously unaware tension
- Somatic awareness
- Bodily knowledge
- Somatic learning
- Corporeal localization
- Somatic epistemology
- Embodied knowing
- Somatic comprehension

**Personal Experiential Theme 1B: The Body That Knows**  *Learning as corporeal transformation*

**Cluster 3: Shared Embodiment**

- Shared movement
- Synchrony
- Intercorporeality
- Boundary dissolution
- Extended embodiment
- Collective agency
- Shared intentionality
- Tool-like extension
- Distributed cognition
- Intercorporeal transmission

**Personal Experiential Theme 1C: We-Body**  *The experience of shared bodily existence*

**Cluster 4: Temporal Alteration**

- High-stakes context
- Moment of difficulty
- Temporal alteration
- Kairos moment
- Post-intervention transformation
- Temporal persistence
- Enduring embodiment
- Phantom presence
- Internalized mentor

**Personal Experiential Theme 1D: Time Embodied** *Temporal dimensions of bodily learning*

**Cluster 5: Beyond Language**

- Non-verbal intervention
- Silence as pedagogy
- Beyond language
- Pre-verbal learning
- Contrast with formal learning
- Analogy

**Personal Experiential Theme 1E: The Unspoken Curriculum**  *Learning that exceeds linguistic representation*

**From Personal Experiential Themes to a Preliminary Case-Level Synthesis (P4)**

These five Personal Experiential Themes were then synthesized into a preliminary case-level synthesis for this participant:

**Preliminary Case-Level Synthesis for P4:**
**"The Pedagogy of Presence"** *Learning through shared corporeal existence in moments of clinical intensity, where knowledge passes between bodies without words, transforming the learner's embodied being and creating enduring somatic memory.*

**Part C: Cross-Case Analysis**

**Comparing Personal Experiential Themes Across Participants**

The same process was repeated for all participants. Below is a comparison of how the "embodied learning" dimension appeared across cases:

| Participant | Key Embodied Learning Descriptions | Personal Experiential Themes |
| --- | --- | --- |
| **P1 (Pediatrics)** | "I caught myself standing exactly like her at the bedside... My body learned her stance of attentive listening before my mind recognized it." | Kinesthetic mirroring; pre-cognitive imitation; postural inheritance |
| **P2 (Pediatrics)** | "During a resuscitation, our breathing synchronized. I knew what he needed before he asked." | Intercorporeal synchrony; shared intentionality; non-verbal coordination |
| **P3 (Gynecology)** | "When she places her hand on my back during a difficult delivery, I feel calmer. Her body communicates confidence to mine." | Affective transmission; somatic reassurance; touch as emotional regulation |
| **P4 (Surgery)** | "My hands became extensions of his judgment... The knowledge is in my body." | Haptic learning; embodied judgment; somatic memory |
| **P5 (Orthopedics)** | "I can feel the way he held the drill the angle, the pressure. My hands remember what his showed them." | Procedural embodiment; tactile memory; skill sedimentation |
| **P6 (Anesthesia)** | "When I'm intubating, I sometimes feel his presence behind me, even though he's not there. My body expects his hands." | Phantom mentor; embodied expectation; internalized presence |
| **P7 (Internal Med)** | "His sigh taught me when to slow down. I didn't learn that from words I learned it from the sound of his breath." | Auditory embodiment; affective attunement; tempo learning |
| **P8 (Family Med)** | "I notice myself using the same gesture he used when listening this slight tilt of the head. It wasn't planned; it just appeared." | Gestural inheritance; unconscious incorporation |
| **P9 (Surgery)** | "When I'm anxious, I remember how still she stood during crises. My body tries to find that stillness." | Compartment modeling; somatic regulation; embodied coping |
| **P10 (Pediatrics)** | "The way he touches children so gentle, so present. I feel that in my own hands now when I examine kids." | Relational touch; empathetic embodiment; care transmission |

**Synthesizing Across Cases: The Emergence of "Embodied Epistemology"**

Through iterative comparison across all ten participants, the preliminary case-level synthesis evolved into a more robust, theoretically informed conceptualization:

**Initial synthesis:** "Learning Through the Body"
**Refined synthesis:** "Corporeal Transmission of Clinical Knowing"
**Final Group Experiential Theme (GET):** **"Embodied Epistemology: The Corporeal Transmission of Clinical Knowing"**

This final formulation captures:

- The *epistemic* dimension (that bodies *know*)
- The *transmission* dimension (that this knowing passes between persons)
- The *corporeal* dimension (that this occurs through and in bodies)
- The *phenomenological* dimension (how this is *lived* by residents

**Part D: Theoretical Integration**

**Illuminating Data with Phenomenological Concepts**

The data was brought into dialogue with phenomenological theory, particularly Merleau-Ponty's concepts, to deepen interpretation:

| Participant Account | Phenomenological Concept | Interpretive Insight |
| --- | --- | --- |
| "My hands weren't mine alone anymore" (P4) | Intercorporeality (Merleau-Ponty, 1962) | The body schema extends to incorporate the other's body in joint action |
| "My body learned her stance... before my mind recognized it" (P1) | Pre-reflective consciousness; corporeal intentionality | Learning occurs first at the bodily level, only later becoming conscious |
| "Our breathing synchronized during resuscitation" (P2) | Pairing; intercorporeal coupling | Bodies entrain to each other in shared intentional projects |
| "The knowledge is in my body" (P4) | Somatic sedimentation; habit-body | Skills become incorporated into the body's permanent repertoire |
| "I feel his hands over mine even now" (P4) | Phantom presence; the invisible other | The mentor persists as embodied absence, an internalized corporeal presence |
| "His sigh taught me when to slow down" (P7) | Affective intentionality; emotional transmission | Emotions are not private but pass between bodies atmospherically |

**Theoretical Refinement of the Theme**

Drawing on Merleau-Ponty's (1962, 1968) work, we refined "Embodied Epistemology" through several interconnected concepts:

**1. The Lived Body (Corps Vécu)**
Rather than an object among objects, the body is our point of view on the world. Residents' accounts reveal that clinical learning is not something that happens *to* the body but *through* the body as subject.

**2. The Habit-Body**
Skills become sedimented in the body as permanent acquisitions. P4's "my hands *knew* something they hadn't known before" exemplifies how procedural knowledge becomes incorporated into bodily being.

**3. Intercorporeality**
Merleau-Ponty's late work emphasizes that bodies exist in reciprocal relation, each responding to and incorporating the other. The synchrony described by P2 and the shared movement described by P4 exemplify this intercorporeal dimension.

**4. The Chiasm**
The reversible relation between perceiver and perceived, toucher and touched. P4's description of feeling "what he was feeling through his hands" suggests a chiasmic intertwining of sensory capacities.

**5. Flesh (La Chair)**
The elemental medium of bodily existence. The warmth, pressure, and rhythm described by participants suggest a shared corporeal medium through which clinical wisdom flows.

**Part E: Reflexivity Notes Throughout Analysis**

**Excerpts from Researcher's Reflexive Journal**

**Entry Date: November 15, 2024**

*After coding P4's interview today, I'm struck by how my own surgical training is shaping my interpretation. I find myself nodding vigorously at his description of hands being guided I remember that experience vividly from my own residency. Is this resonance helping me understand, or blinding me to aspects I take for granted? I need to explicitly bracket my assumption that haptic learning is universally positive. What about moments when touch is experienced as violation or control? I must actively look for disconfirming evidence.*

**Entry Date: December 3, 2024**

*In team meeting today, ATB challenged my emerging theme of "intercorporeality." He asked: "Are you imposing Merleau-Ponty on the data, or is the data calling for Merleau-Ponty?" Good question. I went back to P2's account of synchronized breathing during resuscitation. The data itself uses language of "we," "together," "shared." The theory illuminates what's already present. But I need to ensure I'm not selecting only data that fits. I'll systematically search for accounts that don't fit the intercorporeal frame.*

**Entry Date: January 22, 2025**

*Found important counter-evidence today. P9 described a mentor whose touch felt controlling, not pedagogical. "He would grab my hands and force them, not guide them. I learned from that too I learned how NOT to teach." This complicates the narrative. Embodied learning isn't always positive. The body can learn fear, hesitation, violation. Need to ensure the final theme includes this complexity that embodied epistemology includes both affirming and traumatic transmissions.*

**Entry Date: February 18, 2025**

*Returned to P1's transcript with fresh eyes after reading van Manen on the difference between "lived body" and "physical body." P1's description of "catching myself standing exactly like her" isn't about the physical body as object but about the lived body as intentional presence. She didn't decide to imitate; her body spontaneously oriented itself toward the world in her mentor's way of orienting. That's the lived body learning new ways of being-in-the-world.*

**Entry Date: March 5, 2025**

*Shared preliminary findings with three participants for member checking. P4's response to the "Embodied Epistemology" theme: "Yes, that's exactly it. You've named something I felt but couldn't say." P1 added: "I'd never thought about my body learning, but now I notice it everywhere. Yesterday I caught myself using her exact tone of voice with a frightened child." Their confirmation feels significant, though I know member checking isn't about verification but about deepening interpretation. P9, who described negative touch, said: "It's important that you included both kinds. Because our bodies learn from the bad as much as the good, just in different ways."*

**Part F: Thematic Evolution Documentation**

**Visual Representation of Thematic Development**

text

Stage 1: Initial Codes (150+ codes across all participants)

↓

Stage 2: Clustered Codes (30-40 clusters per participant)

↓

Stage 3: Personal Experiential Themes (5-8 per participant)

↓

Stage 4: Cross-Case Synthesis (12 candidate themes across all participants)

↓

Stage 5: Group Experiential Theme Development (4 final themes)

↓

Stage 6: Theoretical Refinement (Integration with phenomenological concepts)

↓

Stage 7: Final Theme Articulation with Subthemes

| Stage | Theme Formulation | Key Insights Added |
| --- | --- | --- |
| **Initial coding** | "Learning through body" | Participants describe bodily dimensions of learning |
| **Personal Experiential Themes (P4)** | "The Pedagogy of Presence" | Focus on haptic learning and intercorporeality |
| **Cross-case comparison** | "Corporeal Transmission" | Recognition that this pattern appears across all participants |
| **First synthesis** | "Bodily Ways of Knowing" | Introduction of epistemological language |
| **Theoretical integration** | "Embodied Epistemology" | Merleau-Ponty's concept of lived body provides theoretical grounding |
| **Subtheme development** | Haptic, kinesthetic, affective, intercorporeal dimensions | Identification of distinct but related embodied modalities |
| **Final articulation** | "Embodied Epistemology: The Corporeal Transmission of Clinical Knowing" with four subthemes | Fully developed theme integrating all analytical levels |

**Evolution of "Embodied Epistemology" Through Analytical Stages**

**Part G: Final Thematic Structure with Supporting Data**

**Group Experiential Theme 1: Embodied Epistemology**

**Definition:** The phenomenon wherein clinical knowledge, values, and ways of being are transmitted through shared corporeal presence, engaging multiple sensory modalities and resulting in somatic sedimentation that persists beyond conscious recall.

**Subtheme 1.1: Haptic Learning Knowledge Through Touch**

| Participant | Quotation | Phenomenological Interpretation |
| --- | --- | --- |
| P4 | "When he places his hands over mine during a procedure, I feel the pressure, the angle, the rhythm. That touch teaches more than any lecture." | Tactile pedagogy; haptic transmission of procedural knowledge |
| P5 | "I can feel the way he held the drill the angle, the pressure. My hands remember what his showed them." | Somatic memory; skill sedimentation in the body |
| P9 | "He would grab my hands and force them, not guide them. I learned from that too I learned how NOT to teach." | Negative haptic learning; traumatic embodiment |

**Subtheme 1.2: Kinesthetic Mirroring The Body That Remembers**

| Participant | Quotation | Phenomenological Interpretation |
| --- | --- | --- |
| P1 | "I caught myself standing exactly like her at the bedside... My body learned her stance of attentive listening before my mind recognized it." | Pre-cognitive imitation; postural inheritance |
| P8 | "I notice myself using the same gesture he used when listening this slight tilt of the head. It wasn't planned; it just appeared." | Gestural incorporation; unconscious modeling |
| P10 | "The way he touches children so gentle, so present. I feel that in my own hands now when I examine kids." | Relational touch transmission; care embodiment |

**Subtheme 1.3: Affective Atmospheres Catching Emotional Dispositions**

| Participant | Quotation | Phenomenological Interpretation |
| --- | --- | --- |
| P7 | "When he enters the ward, there's a calm that spreads. My own anxiety settles. I've started noticing how my presence affects the team." | Affective transmission; emotional resonance |
| P3 | "When she places her hand on my back during a difficult delivery, I feel calmer. Her body communicates confidence to mine." | Somatic reassurance; touch as emotional regulation |
| P9 | "When I'm anxious, I remember how still she stood during crises. My body tries to find that stillness." | Compartment modeling; embodied coping |

**Subtheme 1.4: Intercorporeal Synchrony Shared Bodily Intentionality**

| Participant | Quotation | Phenomenological Interpretation |
| --- | --- | --- |
| P2 | "During a resuscitation, our breathing synchronized. I knew what he needed before he asked not because I was thinking, but because my body was somehow moving with his." | Intercorporeal coupling; shared intentionality |
| P4 | "For a few seconds, we were moving together. My hands weren't mine alone anymore; they were somehow his hands too, or maybe our hands together." | Boundary dissolution; collective agency |
| P6 | "When I'm intubating, I sometimes feel his presence behind me, even though he's not there. My body expects his hands." | Phantom mentor; embodied internalization |

**Part H: Audit Trail Documentation**

**Analytical Decision Log**

| Date | Decision | Rationale |
| --- | --- | --- |
| 10/15/24 | Conduct second interviews 3-4 months after first | Allow time for journaling and reflection; enable deeper exploration of emerging themes |
| 11/30/24 | Include negative cases in embodied learning theme | P9's account of controlling touch revealed that embodiment includes both positive and traumatic transmissions |
| 12/12/24 | Adopt van Manen's lifeworld existentials as organizing framework | Provides systematic phenomenological structure while allowing inductive themes to emerge |
| 1/18/25 | Integrate Foucauldian analysis alongside phenomenology | Participants' accounts revealed power dimensions that phenomenological analysis alone could not fully address |
| 2/5/25 | Return to Merleau-Ponty's *Visible and Invisible* | P4's description of shared movement demanded theoretical resources beyond *Phenomenology of Perception* |
| 2/28/25 | Refine "Embodied Epistemology" from four to five subthemes, then back to four | Initial five subthemes collapsed when "proprioceptive" was integrated into "kinesthetic" as distinct but related |
| 3/10/25 | Member check with three participants | Ensure interpretations resonate with lived experience; refine language of "Relational Dialectics" theme based on feedback |

**Team Meeting Notes (Excerpt)**

**Meeting Date: January 12, 2025**
**Attendees:**

**Topic:** Refining the "Embodied Epistemology" theme

**X:** I'm struggling with whether "affective atmospheres" belongs under embodiment or should be its own theme.

**Y:** What's the phenomenological basis for including it?

**X:** Participants describe emotions as something they catch from mentors—not as private mental states but as something that passes between bodies. P7's description of calm spreading when her attending enters the ward—that's experienced bodily, not cognitively.

**Y:** So emotions are experienced as atmospheric, as something that permeates the shared space and affects bodies?

**X:** Exactly. And P3's description of her mentor's hand on her back communicating confidence that's both touch and emotion together. They're inseparable.

**Y:** Then it belongs in embodiment. But we need to be clear that we're not reducing emotion to physiology. It's about the lived experience of emotional transmission through bodily presence.

**X:** Agreed. I'll refine the subtheme to emphasize the atmospheric, intercorporeal quality of affective transmission.

**Part I: Mapping Themes to Participant Accounts**

**Presence of Embodied Epistemology Across Participants**

| Participant | Haptic Learning | Kinesthetic Mirroring | Affective Atmospheres | Intercorporeal Synchrony |
| --- | --- | --- | --- | --- |
| P1 | Moderate | Strong | Moderate | Minimal |
| P2 | Minimal | Moderate | Strong | Strong |
| P3 | Strong | Minimal | Strong | Moderate |
| P4 | Strong | Moderate | Moderate | Strong |
| P5 | Strong | Moderate | Minimal | Minimal |
| P6 | Moderate | Minimal | Strong | Moderate |
| P7 | Minimal | Moderate | Strong | Minimal |
| P8 | Minimal | Strong | Moderate | Minimal |
| P9 | Strong (negative) | Moderate | Strong | Minimal |
| P10 | Strong | Strong | Moderate | Moderate |

**Note:** This table demonstrates that while all participants contributed to the Group Experiential Theme, the relative salience of each subtheme varied based on specialty, individual experience, and the nature of modeling relationships.

**Part J: From Themes to Narrative**

**Writing the Phenomenological Text**

The final stage involved transforming analytical themes into evocative phenomenological description. Below is an excerpt from the manuscript showing how the analytical work above was rendered into narrative form:

**From the Manuscript (Section 3.1):**

"Residents consistently described learning through role modeling as fundamentally embodied, engaging multiple sensory modalities beyond visual observation. The pedagogical power of tactile experience emerged repeatedly, particularly in procedural disciplines. As P4 noted regarding surgical mentoring: 'When he places his hands over mine during a procedure, I feel the pressure, the angle, the rhythm. That touch teaches more than any lecture.' This haptic dimension created what might be called a 'muscle memory of care' a corporeal sedimentation of clinical wisdom that persists beyond conscious recall.

Beyond explicit touch, residents described unconscious processes of kinesthetic mirroring that preceded cognitive recognition. Through repeated co-presence with role models, they found themselves adopting postures, gestures, and rhythms of movement characteristic of their mentors. P1 observed: 'I caught myself standing exactly like her at the bedside... My body learned her stance of attentive listening before my mind recognized it.' This suggests that professional comportment the characteristic ways clinicians inhabit their bodies in clinical space is transmitted through embodied resonance rather than explicit instruction.

Participants also described 'catching' emotional dispositions from role models, experiencing the clinical environment as suffused with affective qualities that shaped their own emotional experience and regulation. P7 explained: 'When he enters the ward, there's a calm that spreads. My own anxiety settles. I've started noticing how my presence affects the team when I'm rushed and anxious, everyone feels it. So I try to carry something of his calm into the room.'

The most profound embodied experiences involved moments of what Merleau-Ponty terms 'intercorporeality' a fusion of bodily intentionality wherein residents experienced themselves as temporarily extended into the body of the other. P2 described: 'During a resuscitation, our breathing synchronized. I knew what he needed before he asked not because I was thinking, but because my body was somehow moving with his. We weren't two separate people anymore; we were one organism responding to this child's body in crisis.'"

**Part K: Verification of Interpretive Adequacy**

**Criteria for Trustworthiness in Phenomenological Analysis**

| Criterion | How Addressed | Evidence |
| --- | --- | --- |
| **Sensitivity to context** | Attention to Ethiopian institutional and cultural context; engagement with relevant literature | Section 1.3; detailed contextual description throughout |
| **Commitment and rigor** | Prolonged engagement (9 months); multiple data sources; systematic analysis | Section 2.3; comprehensive coding; audit trail |
| **Transparency and coherence** | Clear presentation of methods; audit trail; reflexive documentation | This Supplementary File; Section 2.6 |
| **Impact and importance** | Contribution to theory and practice; relevance to resource-limited settings | Section 4.5; Section 5 |
| **Iterative process** | Movement between data and interpretation; team discussions; member checking | Team meeting notes; member checking documentation |

**Part L: Concluding Reflections**

This analysis trail documents the hermeneutic journey from raw data to final interpretation. It demonstrates that the theme Embodied Epistemology did not emerge fully formed but evolved through sustained engagement with participants' accounts, dialogue with phenomenological theory, reflexive attention to researcher positionality, and systematic comparison across cases.

The trail also reveals that phenomenological analysis is neither purely inductive (allowing themes to "emerge" untouched by theory) nor purely deductive (imposing pre-existing frameworks on data). Rather, it involves a hermeneutic circle wherein data illuminates theory and theory illuminates data, each transforming the other in an ongoing process of interpretation.

By making this process visible, we hope to demonstrate the trustworthiness of our findings and contribute to methodological transparency in phenomenological medical education research.
