# Supplementary material for: Beyond Observation: A Hermeneutic Phenomenological Study of Role Modeling Experiences Among Clinical Residents in an Ethiopian Teaching Hospital: A Qualitative Study
Source: Health Sci Rep. 2026 Sep 3;9(9):e73179. doi: 10.1002/hsr2.73179 (PMC13542182; doi:10.1002/hsr2.73179)
Supplement: Supplementary file 4 [file HSR2-9-e73179-s004.docx]

**Supplementary File 2: Interview Protocol and Phenomenological Prompts**

**Part A: Semi-Structured Interview Guides**

**First Round Interview Guide: Exploring Lived Experience of Role Modeling**

**Opening and Orientation (5 minutes)**

Thank you for agreeing to participate in this study. As we discussed, I'm interested in understanding your lived experience of learning from senior clinicians what it's actually like to be in relationships with role models during your residency. There are no right or wrong answers; I'm simply hoping to understand your experience from your perspective.

Before we begin, I want to remind you that everything you share will be kept confidential. You can choose not to answer any question, and you can stop the interview at any time. Do you have any questions before we start?

**Grand Tour Question (Phenomenological Opening)**

*"I'd like you to think back over your residency so far and recall a specific moment when you found yourself learning something significant from a senior colleague a moment that stands out as meaningful. Can you describe that moment in as much detail as possible, as if you were painting a picture of what it was like?"*

**Probes for Depth:**

- Where were you? Can you describe the physical space?
- Who else was present?
- What time of day was it? What was the temporal context?
- What were you noticing in your body at that moment?
- What were you feeling emotionally?
- What about this moment made it significant?

**Lifeworld Existential Prompts**

*Lived Body (Corporeality)*

- "When you were observing/learning from this person, what were you aware of in your own body?"
- "Did you notice any physical sensations, postures, or movements?"
- "Was there anything about your mentor's physical presence that stood out?"
- "Have you ever found your body doing something that you learned from watching them?"

*Lived Time (Temporality)*

- "How did time seem to pass during these modeling moments?"
- "Were there moments when time seemed to slow down or speed up?"
- "How do these experiences connect with your sense of past your training before residency?"
- "How do they connect with your sense of future the kind of physician you're becoming?"

*Lived Space (Spatiality)*

- "Can you describe the spaces where meaningful modeling occurs?"
- "How does the physical environment the ward, OR, clinic affect these experiences?"
- "Are there spaces where modeling feels different than others?"

*Lived Relation (Relationality)*

- "How would you describe the quality of your relationship with this role model?"
- "What was it like to be with them in clinical spaces?"
- "How did you experience the hierarchy between you?"
- "Were there things you could or couldn't say or do in their presence?"

**Positive and Negative Modeling**

- "Can you describe a time when you observed something in a senior colleague that you actively wanted to incorporate into your own practice? What made that experience powerful?"
- "Can you describe a time when you observed something you hoped never to repeat? How did that experience affect you?"
- "How do you navigate the experience of admiring certain aspects of a mentor while rejecting others?"

**Closing Reflection**

- "If you were to choose one image or metaphor that captures what role modeling has been like for you during residency, what would it be?"
- "Is there anything about your experience of role modeling that we haven't discussed that feels important?"

**Second Round Interview Guide: Deepening Interpretation**

*(Conducted 3-4 months after first interview, informed by journal data and preliminary analysis)*

**Opening**

"Since we last spoke, you've been keeping a journal about your modeling experiences, and I've had time to reflect on our conversation. I'd like to explore some themes that emerged and hear more about experiences you've had since then."

**Follow-up on First Interview Themes**

- "Last time you described [specific experience/theme]. Can you tell me more about that dimension of your experience?"
- "Have there been moments since we spoke that resonate with or challenge what you described?"

**Embodied Learning Probes**

- "You mentioned [specific embodied experience—e.g., hands being guided, adopting posture]. Can you walk me through another moment when your body learned from a mentor?"
- "Have you become more aware of your body in clinical situations since our last conversation?"

**Temporal Dimensions**

- "You described moments when 'time stopped' or 'folded.' Have there been other moments like that? What characterizes them?"
- "How do you experience the connection between past mentors, present practice, and future teaching?"

**Power and Resistance**

- "You described navigating hierarchy in various ways. Can you tell me about a time when you disagreed with or resisted something a mentor modeled? How did that feel? What did you do?"
- "Are there things you find yourself saying internally to mentors agreements, arguments, questions?"

**Moral Formation**

- "You described learning ethics through observation. Can you describe a specific moment when you witnessed something that shaped your sense of right and wrong as a physician?"
- "How do you carry those moments with you?"

**Closing**

- "What has it been like to participate in this research and reflect on these experiences?"
- "Is there anything you've realized about role modeling through our conversations?"

**Part B: Phenomenological Prompts for Reflective Journals**

**Journal Instructions for Participants**

*Please write in this journal at least once weekly for the next three months. Each week, choose one of the prompts below that speaks to an experience you've had. Write freely and descriptively, focusing on specific moments rather than general impressions. There are no right or wrong answers the goal is to capture your lived experience in as much detail as possible.*

**Weekly Prompts**

**Week 1: First Encounters**
Describe a moment this week when you became aware of learning something from a senior colleague. What happened? Where were you? Who was present? What did you notice in yourself?

**Week 2: The Body Remembers**
Describe a time when you noticed your body doing something you learned from watching a mentor a posture, a way of moving, a way of touching patients or equipment.

**Week 3: Emotional Resonance**
Describe a moment when you "caught" an emotional state from a mentor calm, anxiety, compassion, frustration. What was that like? How did it affect you?

**Week 4: Space and Place**
Describe a specific clinical space where meaningful modeling occurs for you. What makes this space different from others? How does it feel to be there?

**Week 5: Time and Timing**
Describe an experience of time during a modeling moment. Did it speed up, slow down, stop? How did this affect what you learned?

**Week 6: Dialogue and Silence**
Describe a conversation with a mentor that stood out. What was said? What was left unsaid? How did you feel during and after?

**Week 7: Admiration and Ambivalence**
Describe a moment when you felt both drawn to and critical of something a mentor did. How did you navigate this tension?

**Week 8: Ethics in Action**
Describe a moment when you witnessed a mentor navigating a morally complex situation. What did you observe? What did you learn?

**Week 9: Negative Modeling**
Describe a moment when you observed something in a mentor that you hope never to repeat. How did this experience affect you?

**Week 10: Becoming a Model**
Describe a moment when you became aware that a junior colleague might be learning from observing you. What was that like?

**Week 11: Internal Presence**
Describe a time when you heard a mentor's voice in your head during clinical practice guiding, questioning, supporting.

**Week 12: Reflection and Integration**
Looking back over these three months, what have you learned about how role modeling shapes you? What image or metaphor captures your experience?

**Part C: Observation Guide**

**Clinical Observation Protocol**

**Purpose:** To observe the embodied, spatial, and relational dimensions of clinical teaching and learning environments.

**Observer:** 
**Setting:** _________________________
**Date/Time:** _________________________
**Participants present:** _________________________

**Spatial Arrangements**

- How are bodies arranged in the space? (Who stands where? Proximities/distances?)
- How does movement occur through the space?
- What objects/equipment are present and how are they used?

**Embodied Interactions**

- Postures and gestures of attending physicians
- Postures and gestures of residents
- Moments of physical contact (hands guided, touching patients together)
- Gaze patterns who looks at whom, when, for how long?

**Temporal Dimensions**

- Rhythm and pace of activities
- Moments of stillness or pause
- Transitions between activities

**Affective Atmosphere**

- Emotional tone of interactions
- Moments of tension or ease
- Non-verbal emotional communication

**Verbal Exchanges**

- Teaching moments (explicit instruction)
- Questions and responses
- What is said vs. what is left unsaid

**Power Dynamics**

- How is hierarchy enacted?
- Moments of deference, challenge, or resistance
- Who speaks, who listens, who is silent?

**Researcher Reflexivity Notes**

- What am I noticing in myself during this observation?
- What assumptions am I bringing?
- How might my presence be affecting what I observe?

**Part D: Phenomenological Interview Techniques Reference**

| Technique | Description | Example |
| --- | --- | --- |
| Grand tour question | Open-ended invitation to describe experience | "Tell me about a time when..." |
| Descriptive probing | Eliciting sensory and perceptual details | "What did you see/hear/feel in your body?" |
| Evocative questioning | Inviting reliving of experience | "Take me back to that moment. What's happening?" |
| Clarifying | Ensuring accurate understanding | "When you say 'presence,' what does that mean experientially?" |
| Example seeking | Moving from abstract to concrete | "Can you give me a specific example?" |
| Contrast probing | Exploring differences | "How was that different from other modeling experiences?" |
| Reflecting | Offering interpretations for participant response | "It sounds like you felt both drawn and resistant. Is that accurate?" |
| Silence | Allowing space for reflection | (Comfortable pause) |
| Phenomenological nod | Acknowledging resonance | "Yes, that captures something." |

**Techniques Used to Facilitate Phenomenological Reflection**
